# Coatings on mammalian cells: interfacing cells with their environment

**DOI:** 10.1186/s13036-018-0131-6

**Published:** 2019-01-17

**Authors:** Kara A. Davis, Pei-Jung Wu, Calvin F. Cahall, Cong Li, Anuhya Gottipati, Brad J. Berron

**Affiliations:** Chemical and Materials Engineering, University of Kentucky, 177 FPAT, Lexington, KY 40506-0046 USA

**Keywords:** Mammalian cells, Coatings, Polymers, Cell therapy, Cellular coatings

## Abstract

The research community is intent on harnessing increasingly complex biological building blocks. At present, cells represent a highly functional component for integration into higher order systems. In this review, we discuss the current application space for cellular coating technologies and emphasize the relationship between the target application and coating design. We also discuss how the cell and the coating interact in common analytical techniques, and where caution must be exercised in the interpretation of results. Finally, we look ahead at emerging application areas that are ideal for innovation in cellular coatings. In all, cellular coatings leverage the machinery unique to specific cell types, and the opportunities derived from these hybrid assemblies have yet to be fully realized.

## Background

The peripheral membrane of a cell dictates every aspect of how the cell interacts with its environment. While natural function has evolved over time to address the needs of the organism, the peripheral membrane’s natural function is often insufficient for the precise control of when, how, and where a cell interacts with its environment in emerging biomedical needs. As a result, the peripheral membranes of cells are now being tailored to fit the needs of the specific application space through the addition coatings to the cell’s surface.

Cellular coatings are rapidly finding use in a wide range of biomedical research areas. Encapsulation of islets and other cell tissue began in the 1980s [1–3]. While most of these early methods were shown to effectively encapsulate cellular aggregates, low biocompatibility and undesirable mechanical properties limited their effectiveness. The combined work of Pathak et al., Sawhney et al., and Cruise et al. overcame many these obstacles and expanded the encapsulation field when they effectively encapsulated islets of Langerhans and various cells with poly (ethylene glycol) (PEG) in the early 1990s [4–6]. The PEG encapsulated islets introduced the ability of immunosuppression while maintaining cell viability and allowing selective permeability.

While the study of cellular coatings on islets of Langerhans for diabetes treatment continues [7–9], improved understanding of cellular properties and coating techniques has expanded the application space for cellular coatings. Encapsulation techniques are more sophisticated and allow for individual mammalian cells to be modified with polymers. As varying cell types are modified with the coatings, the application space was able to grow beyond immunosuppression.

**Fig. 1:**
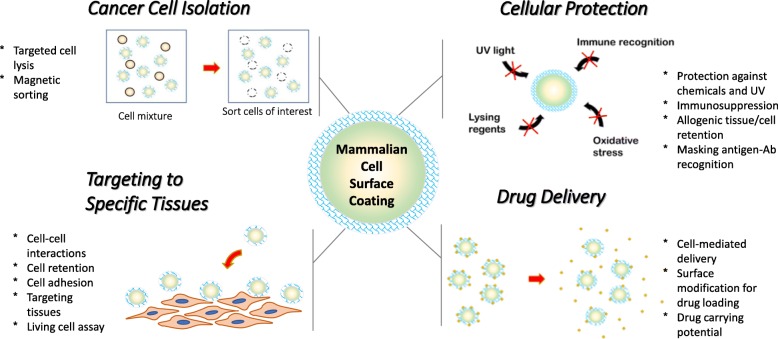
Current applications of mammalian cell surface coatings

In this review we organize the applications of cellular coatings into four subcategories: targeting cells to specific tissues, cell-meditated delivery of drugs, cellular protection in harsh environments, and cancer cell isolation (Fig. 1). We have compiled the most pertinent cell coating literature to give a thorough representation of the cellular coating field. This review also attempts to highlight the various methods used to engineer the cell surface and how these modifications impact the performance of the coated cell.

The scope of this review is limited to coatings of polymers, metals, or ceramic materials to form solid coatings on the surface of individual mammalian cells. In contrast to genetic engineering of cell surface, these robust coatings are capable of driving significant changes to the cell’s natural barrier function and mobility without altering the intrinsic biology of the cell. While there are significant literature of efforts towards the surface engineering of yeast cells [10], the development of mammalian cell coatings provides a more direct connection to biomedical engineering and engineering strategies to impact human health. Finally, this review focuses on the unique functionality of 2D coatings and not on the bulk material techniques prevalent in multicellular encapsulation strategies.

## Application space for cellular coatings

Cellular coatings utilize advancements in surface science to impart the modified cells with unique chemistries and capabilities. In this section, we highlight the most exciting recent developments which leverage the cellular coating of individual mammalian cells. While protection of cells from the immune system and other damaging conditions continues to be explored, cellular coatings also offer the unique ability to drive migration of specific cells to target tissues, deliver payloads across robust biological barriers, and accelerate cellular isolation technologies.

### Adhering cells to specific tissues and substrates

In this section, we highlight the diverse application space for adhesive cell coatings to strengthen cell-cell and cell-tissue interactions. Cell adhesion molecules aid in cell positioning through selective binding to cells and the extracellular matrix. This is most clearly illustrated by the loss of cell-cell adhesion in cancer cells to dislodge a stably-bound cell from the primary tumor site to initiate cancer metastasis [11–16]. The increased mobility caused by the downregulation of cell adhesion molecules permits cancer cells to migrate into the circulatory system, invade neighboring tissues, and develop new tumor sites [17–20]. Cell binding is also critical to the normal function of tissues. For example, an increase in the expression of stromal cell-derived factor 1 (SDF-1) increases the recruitment of therapeutic cardiac stem cells following a heart attack [21, 22]. The direct relationship between adhesion molecule expression and cell localization has motivated the development of artificial cell adhesion technologies for controlling cell position.

#### Targeting inflamed tissues

Inflammation is a natural tissue response to a harmful stimulus. The infiltration of immune cells occurs in concert with dilation of the vasculature and increased vessel permeability. While acute (short term) inflammation is essential for the clearance of a harmful agent, chronic inflammation creates significant health challenges. Increasingly, researchers are using cellular therapies to modify areas of chronic inflammation, and the use of coated cells is an emerging strategy for improving therapeutic cell localization to the inflamed tissue. Many stem cell populations suppress local inflammation through the release of anti-inflammatory factors [23–28]. However, poor stem cell recruitment and retention at the inflamed tissue limits the efficacy of this cell-based therapy. To improve the localization of cells at the therapeutic site, anti-inflammatory stem cells are being coated with inflammation-specific adhesion molecules to mimic the adhesion-driven homing of leukocytes to injuries.

In inflamed tissues, surface carbohydrates on leukocytes bind to selectin and other glycoproteins on the endothelial cell membrane to slow the rolling of the cell along the blood vessel. The decrease of cell velocity aids in leukocyte penetration into the inflamed tissue. Through the accurate recreation of a leukocyte’s inflammation-homing surface, other cell types may be imparted with dramatically improved retention at the site of inflammation. Sarkar et al. created mesenchymal stem cell (MSC) coatings with a sialyl-Lewisx (SLex) ligand that binds to P-selectin on the surface of endothelial cells [29]. They reacted cells with sulfonated-N-hydroxysuccinimide-LC-biotin to display biotin on the MSC surfaces. Subsequent streptavidin exposure leaves excess binding sites for the indirect attachment of biotinylated SLex. They observed a temporary reduction of the accessibility of MSC surface proteins, and the biotin on the stem cell surfaces could be retained up to 1 week without changing cell viability or proliferation. The increased adhesion of SLex-loaded MSCs on P-selectin functionalized surfaces was evaluated by a microfluidic shear assay. The retention of SLex-coated MSCs was increased over uncoated MSCs on the P-selectin surface under identical shear stresses. Under rolling conditions, the significant decrease of the velocity of SLex-labelled cells across P-selectin surfaces was supportive of increased retention at inflammation-adjacent regions of the endothelium.

An in vivo mouse model was utilized to study cell rolling effects in blood flow. The SLex-modified MSC movement through circulation was observed with intravital confocal microscopy of an inflamed ear. The reduced cell rolling speed of SLex-coated MSCs along inner vessel walls indicated strong SLex-selectin binding interaction. The amount of SLex-labelled MSCs at the site of inflamed tissue was significantly higher than that of uncoated MSCs, indicating the importance of SLex surface modification to enhance cell rolling and improve stem cell targeting to the inflamed tissue. The idea of using surface-modified stem cells to mimic leukocyte migration and adhesion on the tissue of interest has also been described by Kong’s recent use of coatings of vascular binding peptides which bind to vascular cell adhesion molecules (VCAM) on the endothelial surface [30]. They used the kinetic models to describe the dependence of peptide-VCAM affinity and ligand surface density on the homing of peptide-engineered stem cells to inflamed or diseased tissues.

**Fig. 2:**
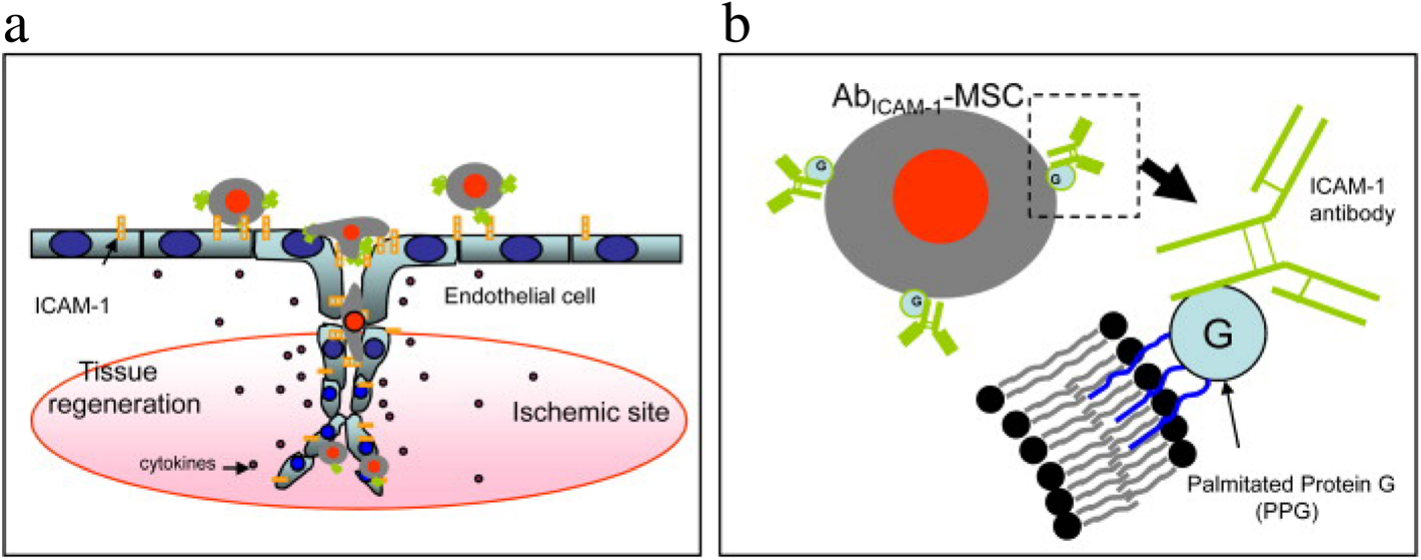
(**a**) ICAM1-antibody coated MSC homing to ischemic site. (**b**) The lipid palmitated protein G conjugation of ICAM1 antibodies to the MSC surfaces. Reprinted from [31], Copyright 2009, with permission from Elsevier

In addition to natural cell adhesion molecules and peptides, antibody-antigen adhesion has been leveraged to improve binding to the inflamed endothelium. Intercellular cell adhesion molecule-1 (ICAM1), vascular cell adhesion molecule 1 (VCAM1), and mucosal addressin cell adhesion molecule 1 (MAdCAM1) expression on endothelial cells is up-regulated with inflammation to promote leucocyte rolling and migration. Coatings of antibodies against ICAM1 have been successful in the targeting of MSCs to inflammation (Fig. 2a) [31]. Ko et al. coated antibodies against ICAM1 on the surface of anchored MSCs (Fig. 2b). The retention of antibody coated MSCs on human umbilical vein endothelial cells (HUVECs) was quantified following exposure to in vitro microfluidic shear. The majority of anti-ICAM1-coated MSCs were retained on the surface of HUVECs, while there was extremely low cell retention in both unmodified MSCs and isotype control antibody-coated MSCs.

**Fig. 3:**
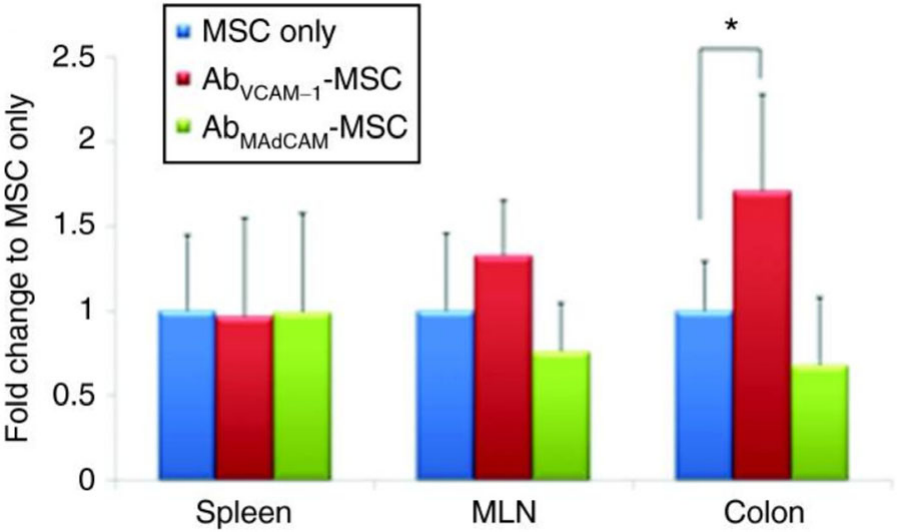
Enhanced MSC accumulation at targeted MLN and colon regions by the VCAM-1-Ab coated MSC delivery. Reprinted from [32], Copyright 2010, with permission from American Society of Gene & Cell Therapy

Surface-modified stem cells have also been utilized to other inflammatory diseases, including inflammatory bowel disease (IBD). Endothelial dysfunction is identified as a pathogenic factor of IBD, therefore targeting the endothelium for stem cell delivery is a logical strategy for IBD therapy. MSCs mediate T cell responses and restore the natural function of IBD tissues in vivo [32]. Ko et al. developed antibody-coated MSCs for the treatment of inflamed colon tissues and inflamed mesenteric lymph nodes (MLN) an in vivo IBD mouse model. They targeted the inflamed tissue using VCAM-1 or MAdCAM-1. Antibodies against VCAM-1 or MAdCAM1 were attached to the peripheral membrane of MSCs. Antibody coated MSCs successfully localized to the target colon tissues and MLN when compared to isotype control cells, and coatings against VCAM-1 showed the strongest effect (Fig. 3). The coated cells remained functional, and the coated cells suppressed T cell proliferation to a greater extent than unmodified MSC groups.

#### Targeting myocardial infarcts

The blockage of blood flow in arteries causes a scarring response which stiffens local tissue in and decreases heart function. Local implantation of MSCs and cardiac stem cells are promising approaches to modulate the inflammatory environment to minimize scarring and support restoration of contractility. Critically, the majority of the injected therapeutic cells are not retained at the site of injury. Several cellular coatings strategies have been developed to increase therapeutic cell retention at the infarct site and improve heart function.

Ferromagnetic cell coatings have potential uses including cellular targeting, tracking, and imaging. Santoso et al. coated cardiac stem cells with an FDA-approved material containing iron oxide nanoparticles loaded with ferumoxytol, heparin, and protamine (FHP) for targeting the cells to an infarcted myocardium [33]. As an added benefit, this magnetic FHP coating is also effective for magnetic cellular tracking of coated cells [34]. External magnetic fields are manipulated to control the position of the FHP-loaded coated cells in an in vivo rat model. Compared with non-magnetically positioned FHP-loaded cells, greater quantities of the magnetically positioned cells were retained at the infarct site [35]. Additionally, the magnetically positioned group had a smaller infarct scar size, thicker myocardium wall, improved ventricular structure, and increased capillary density.

Another coating strategy to enhance cell homing to diseased myocardium leverages the overexpression of homing factors highly expressed on ischemic myocardium, including stromal cell-derived factor 1 (SDF-1). CXCR4 is the natural binding partner for SDF-1, and this interaction is responsible for physiological stem cell recruitment and retention. Cell coatings were designed to present higher levels of CXCR4 to increase the rate of migration toward high levels of SDF-1 [36]. These coatings doubled the in vitro rate of stem cell migration toward a simulated infarcted heart region in a transwell migration assay, demonstrating the potential for similar coatings in future in vivo repair studies.

#### Adhesion of cells to functionalized surfaces and cells in vitro

High throughput, living cell studies are central to the analysis of heterogeneous cell populations. While flow cytometry is well suited for the high throughput analysis of suspended cells, many assays necessitate the cells to be adhered to a solid substrate for image-based observation of signaling, cellular metabolism, or cell interactions [37–39]. Current technologies to attach cells onto a solid surface are mostly applied in adherent cell types. Lacking the expression of adhesion proteins on the cell membrane, suspension cells do not readily attach to most surfaces. Iwasaki et al. demonstrated the anchorage of biotinylated, non-adherent cells on an avidin-patterned substrate [40]. They metabolically tailored human promyelocytic leukemia cells (HL-60) to express methacryoyl modified cell surface carbohydrates, and a thiol-ene reaction was used to graft biotin to the engineered HL-60 cells. These biotin coated HL60 cells adhered specifically to an array of avidin to generate organized cell arrays of suspension cultured cells. Similarly, Kim et al. patterned non-adherent cells into microarrays by coating cells with biotin and creating an adhesive region on PDMS using polymer-on-polymer stamping [41]. Their stamping approach created high fidelity arrays of B cells for high throughput analysis of cell function.

**Fig. 4:**
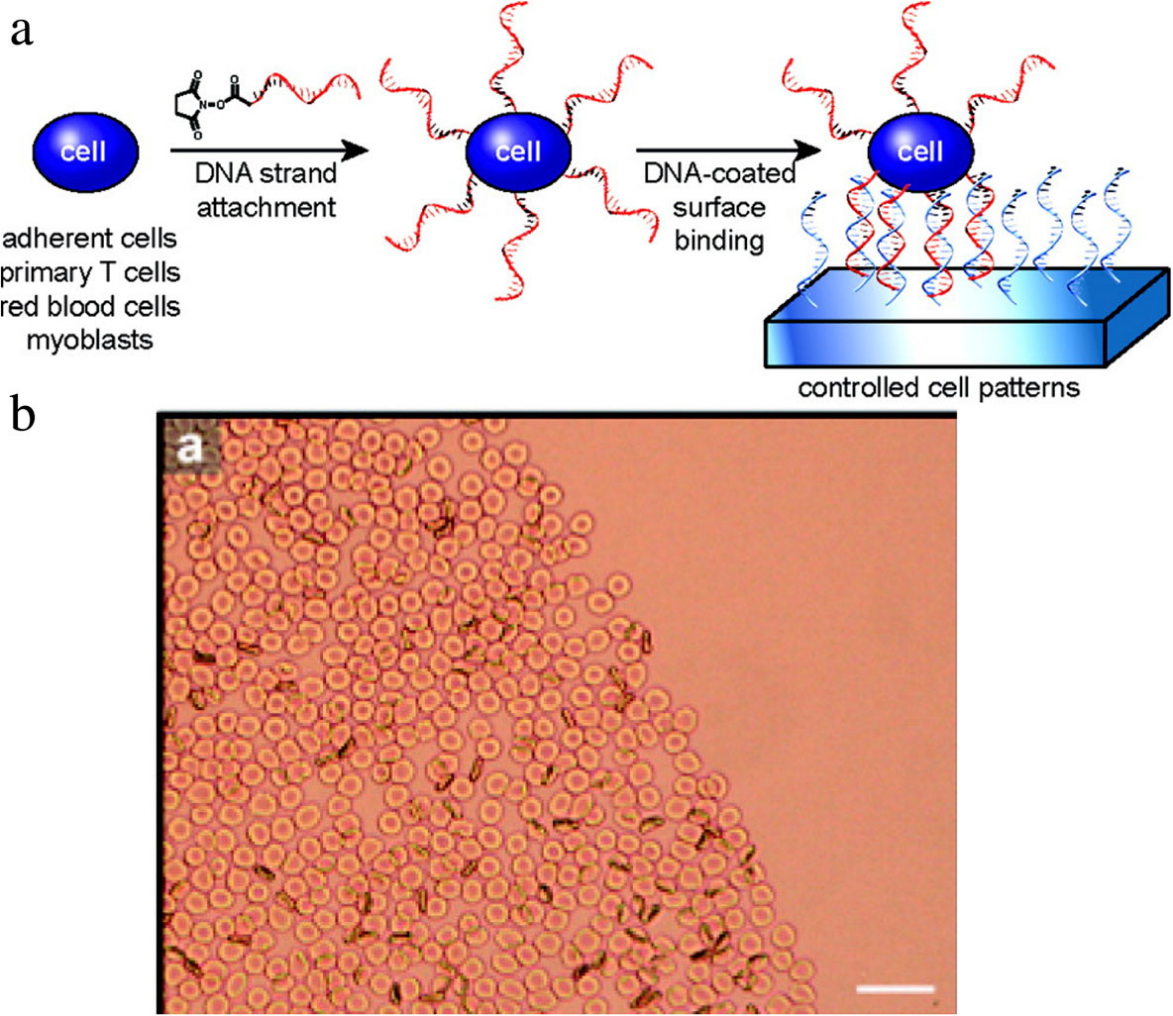
(**a**) The attachment of DNA-coated cells to complementary DNA surfaces. (**b**) The DNA-modified cells are localized to patterned region. Reprinted with permission from Langmuir [42], Copyright 2009, American Chemical Society

Cell arrays are a new approach to study the cell morphology change, cell differentiation or tissue generation within a given spatial arrangement. In one strategy, cell surfaces are coated with single-strand DNA, and these coatings promote specific adhesion of cells to patterned surfaces of complementary DNA sequences (Fig. 4a) [42]. The compatibility of this patterning approach was demonstrated with adherent and nonadherent cell types, including MSCF7, Jurkat, red blood cells, CD4^+^ helper T cells, and cardiac myoblast cells. The two-dimensional patterning of coated cardiac myoblasts displayed a pattern dependence of cell differentiation (Fig. 4b), and it is expected these arrays of coated cells will guide future fundamental studies of tissue assembly, cell behavior, interaction, or tissue dynamics.

In vitro assemblies of multiple cell types are useful for the reconstruction of complex tissue scaffolds. In some cases, cell-cell adhesion in the ECM is required for the regulation of cellular metabolism, cell differentiation, and cell proliferation in tissues [43, 44]. In other cases, the loss of cell interaction reduces adverse effects of many specific cellular activities. As a result, cell coatings for reversible cell-cell interactions facilitates the analysis of many intracellular phenomena [45–48]. Luo et al. created a photo-cleavable cell adhesive strategy by modifying cell membranes with photo-sensitive substances using a liposome fusion approach [47]. Cell interconnection, which leads to cell aggregation or clusters, was successfully achieved through interfacial oxime click ligation chemistry between Jurkats, and disconnection was triggered by photo-cleavage of the tethers in the cell coatings. This approach was extended to multi-cell assemblies to study the differentiation behavior of hMSCs in the presence of fibroblast. Through interaction between photo-oxyamine-coated fibroblasts and ketone-coated hMSCs, multi-cell co-culture of these two different cell types could be organized into layer-by-layer assemblies. The photo-release of fibroblasts from hMSCs enables the further investigation of hMSC functionality after fibroblast-induced differentiation. The oxyamin-ketone ligation-mediated cell assembly/disassembly system established a bidirectional strategy not only to generate a stable multi-tissue scaffold but also to help the understanding of cell performance after multi-cellular co-culture.

**Fig. 5:**
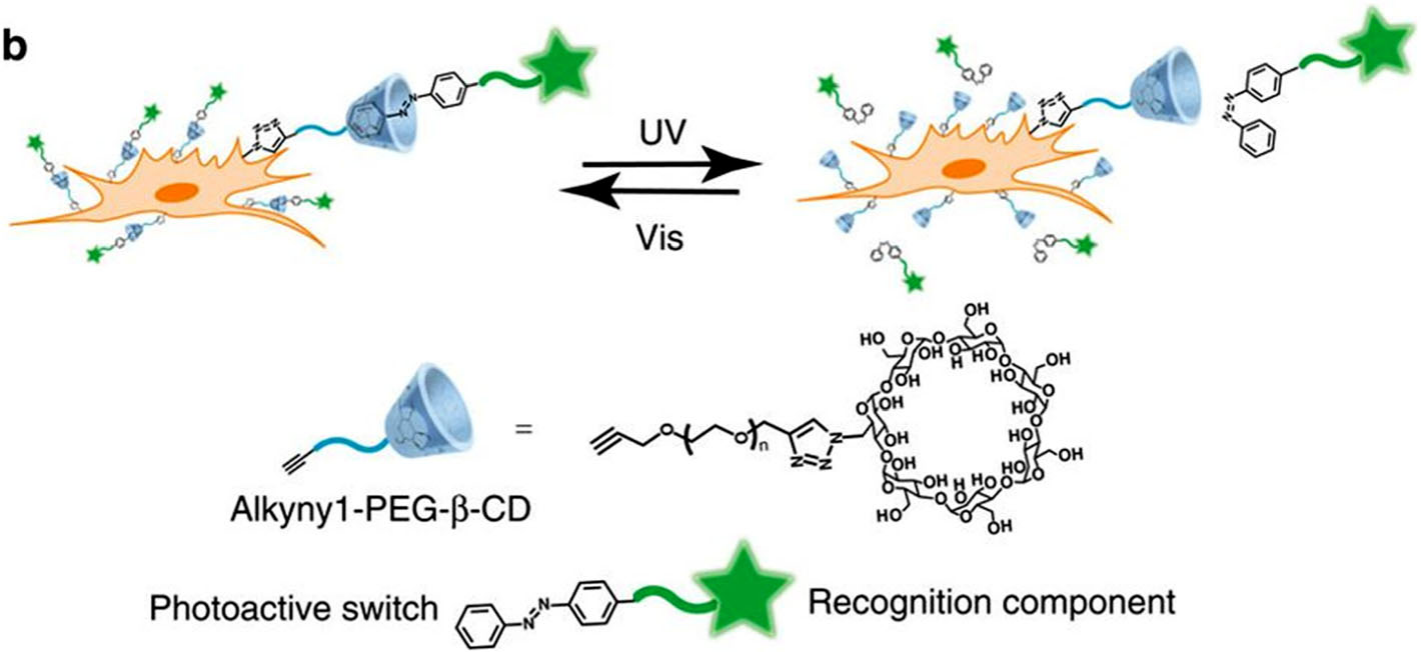
The reversible azobenzene interaction with ß–cyclodextran (CD)-coated cells is under control of light irradiation. Figure reprinted from Nature communications [48]. This work is licensed under a Creative Commons Attribution 4.0 International License. For more information see http://creativecommons.org/licenses/by/4.0/

Cell coatings that incorporate photo-switchable linkages allow a fully-reversible cell-cell interaction [48]. Coatings of ß–cyclodextran (CD) will only react with the trans isomer of azobenzene. When azobenzene is exposed to UV light, the molecule isomerizes to a cis conformation, and will not adhere to ß –CD (Fig. 5). To examine the attachment and detachment efficiency of ß–CD modified cells, cells were seeded on azobenzene surface. ß –CD-coated cells only adhered to azobenzene surfaces in a trans-conformation, and UV-induced cis isomerization resulted in over 80% cell detachment. Additionally, cell coatings of azobenzene groups allowed the reversible attachment with ß –CD coated cells. HeLa cells coated trans-azobenzene groups assemble with ß –CD coated MCF7 cells, and these aggregates disassemble under UV irradiation.

### Cell-meditated delivery of drugs

The human body is a complex bioreactor where an intricate chemical balance is maintained through passive and active transport. Modern drug delivery systems have been successful in leveraging our passive transport mechanisms to deliver drugs through the airways, circulatory system, and diverse tissues. Only recently has the research community started to utilize the active transport mechanisms for cell-directed transport of species in the body. Cell-mediated delivery is a revolutionary approach to exploiting natural cell behaviors, which include migration across biological barriers and targeted homing to tumors or sites of inflammation [49–52]. Cellular coatings are emerging as a promising approach for the delivery of a therapeutic payload to a specific site. Therapeutic cargo is attached to the plasma membrane of tissue-targeting transporter cells to enhance site-specific delivery. Here, we highlight the opportunities enabled by the recent clinical and preclinical studies which coated transporter cells are being used for drug delivery applications.

#### Coated cell mediated delivery for autoimmune/inflammatory diseases

In many autoimmune diseases, immune cells mistakenly identify beneficial cells as a harmful species, resulting in chronic inflammation. In the case of multiple sclerosis (MS), cellular coatings have been designed to introduce immunologically-active substances, preventing the immune cells from attacking the normal nerve cells near the site of the brain and spinal cord [53]. A recent clinical study demonstrated the feasibility of antigen-specific tolerance therapy by intravenously transporting antigen-mimicking cells to T cells [54]. For MS, autologous peripheral blood mononuclear cells are coated with myelin antigens are used to stimulate antigen tolerance through interaction with myelin-specific T cells. For early stage patients treated with myelin coated cells, the vital signs and the blood cell composition of the patients were stable and no relapse occurred within 3 months of treatment.

**Fig. 6:**
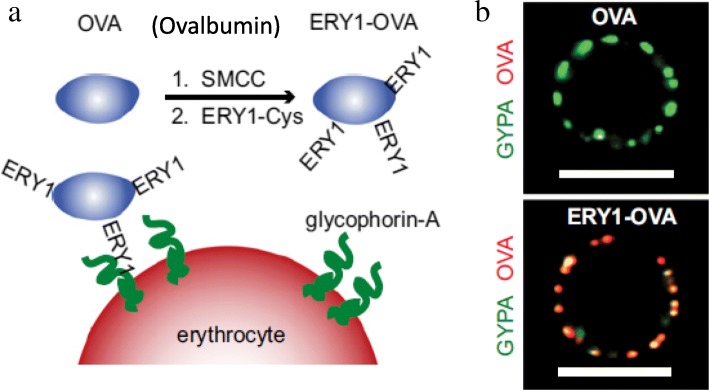
(**a**) Modification of OVA antigens on the erythrocyte surface. (**b**) OVA-conjugated ERY1 peptides (green) interact with erythrocyte-specific protein glycophorin-A (red) on the erythrocyte membrane. Reproduced with permission from PNAS [55]

Similar cell coatings were designed to target and deplete the specific T cell populations responsible for diabetic injury [55]. The surface of erythrocytes were coated with a foreign ovalbumin species (Fig. 6a and b). Following intravenous injection, ovalbumin-coated erythrocytes were disproportionately targeted by the dendritic cells capable of indirect antigen specific tolerance. By also incorporating immune-induced antigens onto the surface of these erythrocytes, the cells promoted targeted depletion of the CD4 and CD8^+^ T cells. These findings were further supported with a type I diabetic mouse model, where antigen-engineered erythrocytes induced clonal depletion of T cells, and the expression of IFN-γ-positive T cells was significantly reduced over ovalbumin-negative controls and ovalbumin alone.

**Fig. 7:**
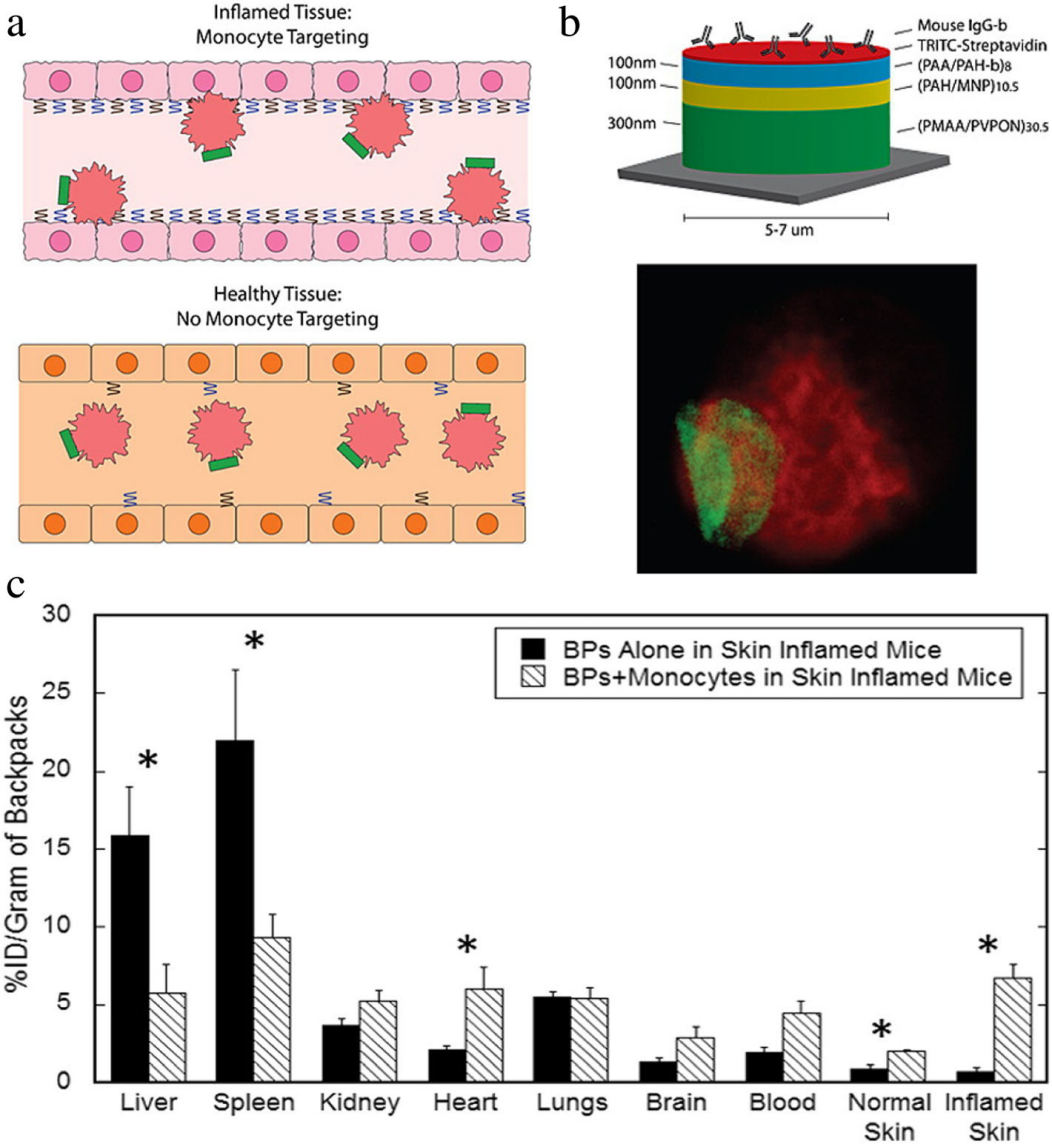
(**a**) Delivery of PEM-backpack-coated monocytes to inflamed tissue. (**b**) Backpack design and adhesion. (**c**) Higher accumulation of modified monocytes in the inflamed mice skin , compared with backpack alone. Reprinted from [59], Copyright 2015, with permission from Elsevier

Multiple cell types naturally migrate to the site of inflammation to serve a reparative function. Drug loaded coatings attached to these naturally-homing cells offer a new opportunity to target therapies to the inflamed region (Fig. 7a). However, the coverage of the entire surface of transporter cells with biomaterials would interfere with the migration and cellular activities of transporter cells throughout the body. Cellular patches were originally proposed by Swiston et al. [56, 57] to partially cover the migratory cell with a surface anchored therapeutic material. In this strategy, polyelectrolyte multilayer (PEM) patches containing a magnetic nanoparticle payload were adhered onto the lymphocyte surface. These T cells were nearly 100% viable following patch modification and retained their migratory capacity with the magnetic PEM patch. This innovative idea of partial surface modification is appropriate to allow either T cell interactions involved in immunological responses or drug carrying potential for cell-based drug delivery.

Additional studies by Doshi et al. [58] found that PEM-modified multilayer patches on the surface of mouse macrophages had no adverse effects on cell viability, migration, or phagocytic activity towards polystyrene spheres. Optimization of PEM patch size, modulus, and shape enabled prolonged patch attachment on the macrophage surface without internalization. In vitro controlled loading of FITC-BSA from polymeric patches supports the feasibility of drug delivery with patch coated cells (Fig. 7b). Patch-coated and uncoated monocytes both undergo stimulation-induced differentiation into macrophages. The targeting of coated monocytes to inflamed skin and lung tissues was supported in mouse models [59]. Higher accumulation of patch-coated monocytes in inflamed tissue over the patch alone, illustrated that coated monocytes retained their ability to penetrate across barriers to target regions of inflammation (Fig. 7c).

#### Coated cell mediated delivery for cancer therapy

The irregular vessel organization, low oxygen supply, and high interstitial fluid pressure of large tumors impedes the delivery of drugs into large malignant tissues. Currently, the majority of tumor-targeting approaches use liposomes, micro- or nanoparticles, and polymers. However, their insufficient penetration into deep tumor tissues and rapid clearance limits therapeutic efficacy. The use of tumoritropic cells as drug carriers is a promising new approach to deeply penetrate tumor tissues and increase drug efficacy [52].

There are now multiple examples of drug-coated T cells used for tumor-specific targeting of a therapy [60, 61]. T cells, B cells, and natural killer cells all have some level of tumoritropic capacity [62–65]. These immune cells migrate across capillaries and blood vessels to tumor sites by targeting the cytokines released by the cancer microenvironment [60, 61]. Irvine et al. coated T cells with liposomes loaded with cytokines promoting help T cell differentiation, growth, and endurance at tumor sites [66, 67]. The nanoparticle (NP) coatings were optimized to minimize any effect on cells’ tumoritropic migration in vitro and in vivo. Later, this team also studied the cell-based delivery of phosphatase-encapsulated lipid nanoparticles to promote T cell expansion through the delivery of T-cell regulating phosphatases [67]. T cells were coated with these therapeutic NPs and used to treat mouse models of human prostate cancer. Mice treated with coated T cells had increased T cell expansion, reduced tumor size, and increased animal survival by 60 days over unmodified T cell groups.

Macrophages are also emerging as a useful tool for cell-mediated penetration into tumor masses. They effectively carry drug-encapsulated liposomes into tumors for simultaneous imaging and therapeutic delivery [68]. After surface modification of doxorubicin (DOX)-loaded liposomes, these drug-loaded macrophages accumulated at the tumor site of an A549 xenograft tumor in a mouse model. 24 h after injection, accumulation of DOX was significantly higher in mice treated with of liposome-DOX-loaded macrophages when compared to PBS, macrophage, DOX and liposome-DOX-injected controls. The weekly intravenous injection of coated macrophages also decreased the 1 month tumor size by around 40% when compared to the DOX control group in the same study.

**Fig. 8:**
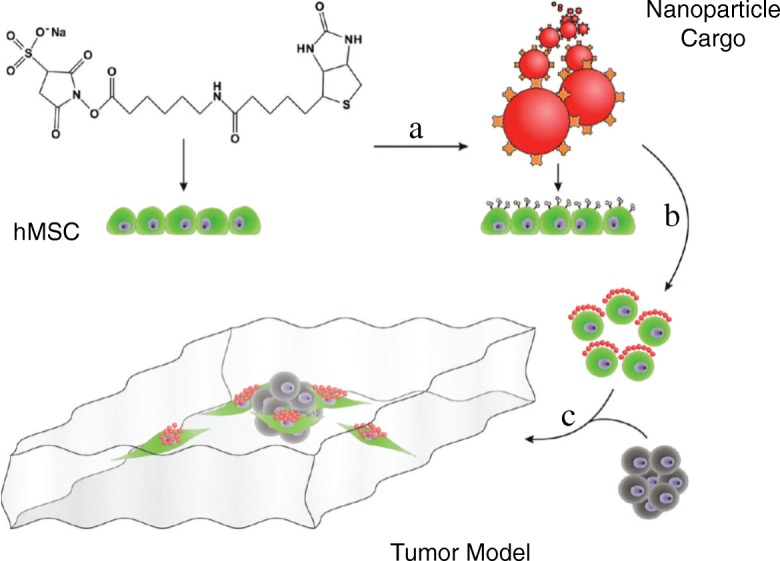
The delivery of NeutrAvidin nanoparticle-coated hMSCs to the HEPG2 tumor spheroid. "Reprinted with permission from [70], Copyright 2010, American Chemical Society"

Many stem cell populations also naturally target tumor sites. Neural stem cells (NSC) are actively studied for the delivery of platinum based therapies to ovarian tumors [69]. NSCs are coated with a nonporous silica that encapsulates platinum-based drugs and has minimal NSC toxicity. These platinum-loaded particles persisted deep inside in solid tumor masses 24 h following intraperitoneal injection into an ovarian cancer mouse model. These findings support NSCs as an alternative cell carrier for drug loaded coatings into the core of tumor masses. Finally, human mesenchymal stem cells (hMSCs) are promising for the delivery of nanoparticle payloads into tumor masses (Fig. 8) [70]. In one study, NP patches were prepared through the attachment of NeutrAvidin-coated NPs to partially modify the surface of covalently biotinylated hMSCs. Coated MSCs targeted HEPG2 tumor spheroids in vitro, and this approach supports the use of nanoparticle-based coatings for cell-mediated tumor targeting.

### Preservation of cellular function in hostile environments

#### Protection against in vivo immune responses

Organ transplant and blood transfusions are common procedures requiring the use of allogenic tissue and cells. However, if this allogenic material displays differing major histocompatibility complex proteins than the host, an immunological response is triggered and the cell or tissue is rejected. T-lymphocytes are primarily responsible for the recognition of allogenic tissue or cells resulting in increased T cells. While conventional therapies to suppress T cell activity are potentially dangerous [71], cellular coatings are a physical approach of camouflaging and protecting the transplanted cell from T cell recognition and rejection. Camouflaging of human peripheral blood mononuclear cells with PEG coatings effectively inhibits the cytokines and Th17 lymphocytes associated with rejection while elevating the Treg cells associated with tolerance in a mouse model [71]. Additionally, in vivo studies showed that after injection of PEGylated donor splenocytes, Treg lymphocytes remained elevated above the levels in naïve mice while Th17 lymhocyte levels remained unchanged 30 days after injection.

Cellular coatings are also used to camouflage transplanted erythrocytes from a patient’s immune system [72–75]. Whole blood and packed red blood cells are commonly used in cases of anemia, trauma, and during surgery for the restoration of blood volume and oxygen carrying capacity. Blood group antigens on erythrocytes are the most common trigger for immunological rejection of blood products [72, 76], and a diverse supply of blood products must be maintained to assure the availability of properly matched blood. An erythrocyte with masked antigens can act as a “universal” blood cell, where only a single blood supply must be maintained.

The design of the cell coating determines the efficacy of antigen masking, and efficacy varies by the targeted antigen. Cyanuric chloride-PEG coatings effectively mask Rh antigens, but they only partially mask A or B antigens [75]. This divergence in efficacy is likely attributed to a deficiency of amino binding sites on A and B antigens or poor access of these antigens for large PEG macromolecules. The use of a 2-iminothiolane (IT) tether can be used to increase binding site accessibility by maleimidophenyl-PEG (Mal-Phe-PEG) through thiol-maleimide chemistry. Mal-Phe-PEG was found to mask anti-C, anti-c, anti-E and anti-e antigens using this approach with an IT extension. However, of greatest clinical significance, A and B antigens are “camouflaged” from the immune system of Mal-Phe-PEG-5000 and Mal-Phe-PEG-20000 (Table 1) [75]. Masking of all ABH/Rh antigens can allow for blood transfusion at a reduced risk of alloimmunization.

**Table 1:** Effectiveness of PEG chain on PEGylated RBCs measured by agglutination (0 indicates no agglutination). Mal = Maleimide; CnCl = Cyanuric chloride. Reproduced with permission from John Wiley and Sons [75]

Modifying PEG Reagent^a^	Agglutination with	
	Anti-A	Anti-D
None	4+	3+
Mal-Phe-PEG-5000	4+	0
Mal-Phe-PEG-10000	2+	0
Mal-Phe-PEG-20000	1+	1+
Mal-Phe-PEG-5000 + 10,000 ^b^	4+	0
Mal-Phe-PEG-5000 + 20,000 ^b^	1+	1+
Mal-Phe-PEG-5000 + 10,000 + 20,000 ^b^	3+	0
Mal-Phe-PEG-5000 and 20,000 ^c^	0	0
CnCl-PEG-5000	4+	1+

^a^All the incubations were carried out in the presence of IT

^b^The PEG reagents of different chain lengths were present together in the reaction mixture

^c^The RBCs were first PEGylated with the Mal-Phe-PEG-5000. After removing the excess reagents by washing, the PEGylated RBCs were reincubated with Mal-Phe-PEG-20000 in the presence of IT

In addition to thiol-maleimide-based PEGylation, other coating chemistries are effective in masking erythrocyte antigens. Wang, et al. used SVA-mPEG to camouflage RhD(−) antigens and studied masking efficacy by monitoring T cell proliferation and differentiation [73]. Polymer grafting to RBCs with RhD antigens effectively suppressed the immune response in PBMC and dendritic cells of RhD-sensitized women. When compared to negative controls (autologous and heterologous ABO-matched RhD erythrocytes), there was no increase in proinflammatory Th17 cells for the erythrocytes coated with mPEG and the anti-inflammatory Treg populations were maintained or increased.

Hyperbranched polyglycerols (HPG) are also effective in camouflaging RBCs from immunological rejection [74]. HPGs have good blood compatibility both in vivo [77] and in vitro [78], and are easily modified before or after covalent bonding to the cell surface [74]. HPG functionalized with succinimidyl succinate (SS) adheres covalently to amines on the cell surface. In a study by Rossi, N.A.A., et al., full RhD antigen masking was achieved for polymers > 56 kDa, and these high HPG molecular weights resulted in better protection than lower molecular weights (33 kDa). The SS-HPG modification did not damage or rupture the cell. Of particular interest, the dendrimer-like structure of HPG also has many excess hydroxyl groups for the potential incorporation of other coating functionality.

#### Protection against hostile in vitro conditions

The vulnerability of mammalian cells makes cell viability a huge obstacle in many applications. Mammalian cells are surrounded by a thin lipid membrane that is highly susceptible to small changes in the surrounding environment. In contrast, many non-mammalian organisms have rigid coatings which protect them in a more diverse range of environments. For example, microbial cells use their cell membrane to protect themselves from harsh environments [79]. Certain bacteria even use their coating to shutdown metabolic activity to survive harmful UV radiation, temperatures, and chemicals [80]. Several research groups are now actively working to create similar protective coatings for mammalian cells, enabling active control over cell activity in a broad range of harsh environments.

**Fig. 9:**
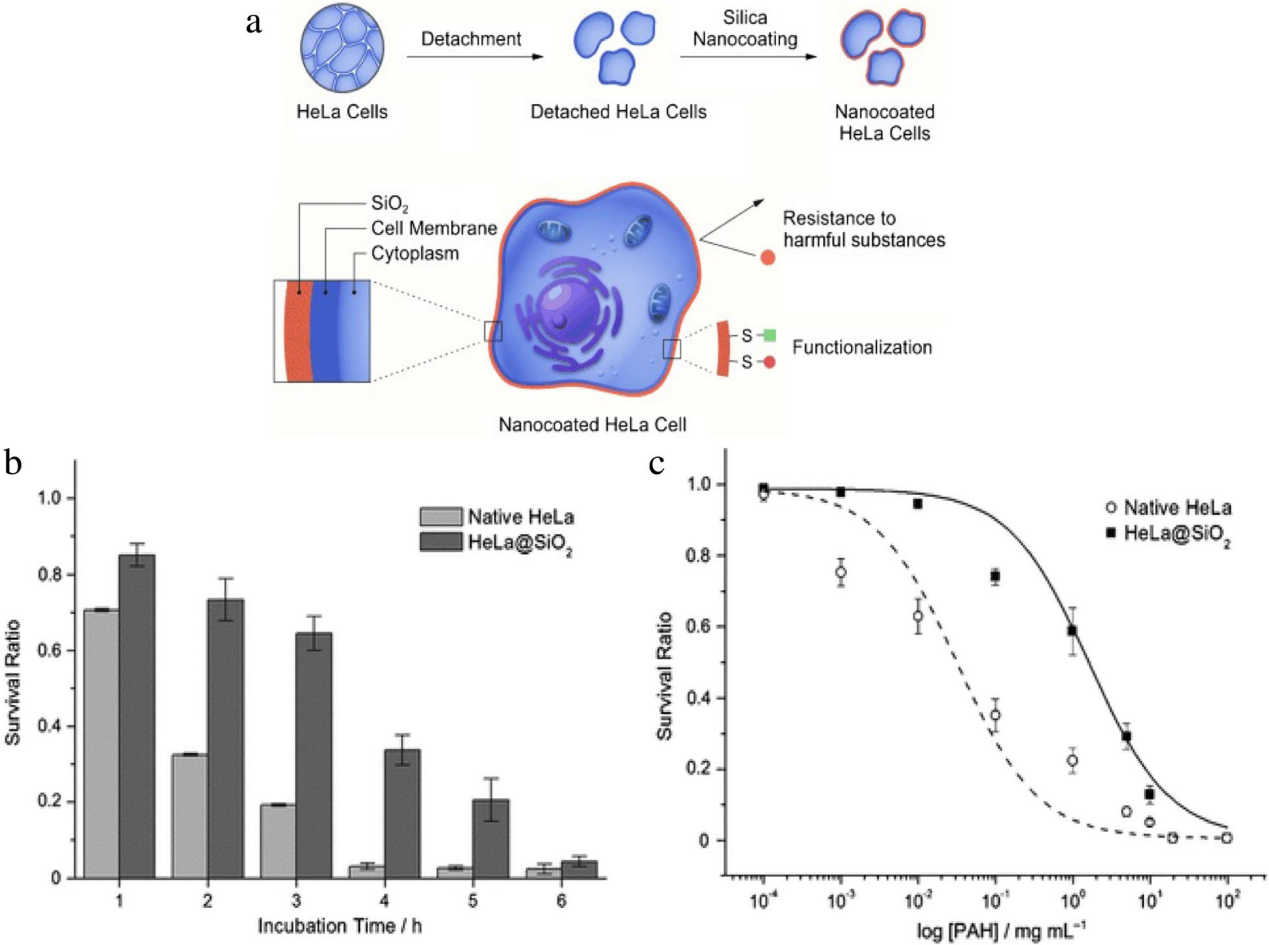
(**a**) Cell Surface modification of HeLa cell with silica nanocoating. (**b**) Protection of HeLa@SiO2 (silica coated) cells versus non-coated HeLa cells to enzymatic trypsin attack. (**c**) Survival ratio of HeLa@SiO2 cells versus uncoated HeLa cells when exposed to various PAH concentrations. Reproduced with permission from John Wiley and Sons [79]

Nanoparticle engineering is a useful tool to protect mammalian cells against foreign toxic substances or stresses [81, 82]. Silica acts to encapsulate some microbial cells as a protective shell. The biosilification of individual mammalian cells was explored by Lee et al. to create protective coatings around cells for improved activity in hostile conditions (Fig. 9) [79]. HeLa, 3 T3 fibroblasts, and Jurkat cells were coated through incubation in poly(ethyleneimine) (PEI) followed by incubation in tetramethyl orthosilicate and 3-mercaptopropyl trimethoxysilane. When compared to unmodified cells, silica coated HeLa cells had higher viability for continued trypsin exposure and high concentrations of the cytotoxic chemical poly(allylamine hydrochloride). Increased viability in these harsh conditions was also achieved for coated fibroblasts and coated Jurkat cells.

Prior work by the same group has showed that the degradable organic-metal nanoshell, produced by the reaction between tannic acid (TA) and ferric ions (Fe), could directly form a protective coating on individual yeast wall [83]. TA, a type of polyphenol, has been known for its reaction with metal ions to form an organic-metal particle complex [84, 85]. According to high protein affinity of TA and TA-Fe reaction, Park et al. generated cytoprotective TA-Fe nanocoatings on the surfaces of various mammalian cells (HeLa, NIH3T3 fibroblast and Jurkat) by simply incubating the mixture of TA and Fe into cell solution. Due to the ability of TA to absorb UV light, the TA-Fe coating can prevent cells from UV irradiation-induced DNA damage. While native cells were ruptured by dehydration or reduced pressure, the results showed that higher UV-irradiation tolerance and higher resistance against cationic polymer (PEI) penetration was achieved for the TA-Fe coated cells compared to the non-coated ones [80]. Additionally, coatings based on this metal-organic complex preserved mammalian cell viability better than the silica coatings [79].

Fully organic coatings also protect mammalian cells against harsh environments. PEGDA coatings protect cells from chemical lysis through exclusion of harsh surfactants. Photopolymerized coatings formed in buffered solutions of 25 wt% PEGDA (Mn 575 or 3500) preserve the viability of encapsulated Jurkat and A549 cells in 5% sodium dodecyl sulfate (SDS) for at least 15 min [86, 87]. When using a photodegradable PEGDA chemistry [88], viable cells are released following SDS exposure to proliferate in vitro [86]. These same coatings protect cells from hypotonic lysis in pure deionized water. Under hypotonic conditions, the coatings mechanically reinforce the cell membrane to prevent rupture of the cell.

Zhang, et al. designed a dextran-based drug eluting conformal coating that was anchored to human embryonic kidney 292 T cells while still maintaining high cell viability [89]. Curcumin was used as a model drug for its anti-oxidant and anti-inflammatory nature which was shown to slowly release over 72 h. The cellular coating was shown to protect the cells from reactive nitrogen and oxygen species in vitro. While results showed the coating system could be clinically beneficial following transplantation, a three-day observation of rhodamine-labeled coatings showed that the majority of coating fluorescence was located in the cytoplasm making it unsuitable for permeant encapsulation.

Oxidative damage to cell membrane proteins is a major cause of shortened circulation time of erythrocytes. As erythrocytes age, cells undergo lipid peroxidation which increases the amount of phosphatidylserine in the outer membrane, aggregation of Band 3 proteins, and early clearance of erythrocytes [90]. TEMPO is a radical scavenger, and cell surface coatings incorporating TEMPO protected erythrocytes in an in vitro model of oxidative stress longer than control coatings without TEMPO. The benefit of TEMPO was found to be concentration dependent, and the incorporation of TEMPO was not cytotoxic. Interestingly, polymer coatings which lack TEMPO also offered some protection against erythrocyte clearance. Addition of the cross-linking agent bis(sulfosuccinimidyl)suberate to covalently bound poly(dimethylacrylamide) minimized the aggregation of Band 3 proteins. Overall, this class of mammalian cell coating retained cell membrane integrity for at least 24 h and erythrocyte aggregation was not observed [90].

### Cell isolation for Cancer diagnostics

Cancer patient survival and quality of life are all strongly correlated with the stage at diagnosis. As a result, analysis of the peripheral blood for signs of cancer is being pursued as an opportunity for routine, minimally invasive cancer screening. The presence of tumor cells in circulation for early stage disease is a potential diagnostic marker residing in an easily accessible tissue. The critical challenge of a blood biopsy for cancer is the isolation of targeted cells from unwanted cells, and the isolation of these living cells is commonly achieved with cell surface modification [87].

Magnetic-Activated Cells Sorting (MACS) is based on forming magnetic coatings on cells and is already widely used in clinical cell sorting [91, 92]. Alternative sorting techniques based on protective cell coatings area also emerging to fill niche applications. MACS utilizes paramagnetic coatings to alter the position of specifically labeled cells. MACS coatings typically consist of labeled magnetic nanoparticles or microparticles that are targeted to specific cells with antibodies or specific protein interactions [93]. Typically, antibody-labeled magnetic beads are used to make antigen-positive cells susceptible to magnetic fields. Cells are then passed through a column surrounded by a magnetic field which pulls magnetically functionalized cells to the column walls. Cells that are not pulled to the wall are collected separately. These coatings are designed as assemblies of individual particles on the cell surface to not interfere with normal cellular processes. In most cases, these coatings are not specifically removed, and their impact on cell function is minimal. In cases where the residual coating is not preferred, many methods for detaching the magnetic particles from the cell have been successful [93].

The performance of these MACS systems is best contrasted with the other dominant cell sorting technology, fluorescence activated cell sorting (FACS). MACS allows for high throughput separations that are not as easily achieved with FACS. The greatest disadvantage to MACS is lower purity than achieved by FACS. In order to combat this, in recent years cells have been passed through the magnetic field twice, each time using a different magnetic tag, to achieve > 90% purities [94]. Most commonly, high purity cell sorting is achieved by implementing magnetic sorting first to perform an enrichment step followed by FACS to achieve the desired high purity. Despite the lower selectivity, many labs will still use MACS because it is much cheaper than using FACS. Specifically at many research universities, FACS user fees are >$100 / hr. (up to 10^8^ cells [95]) whereas the AutoMACS systems only cost $15/sample.

**Fig. 10:**
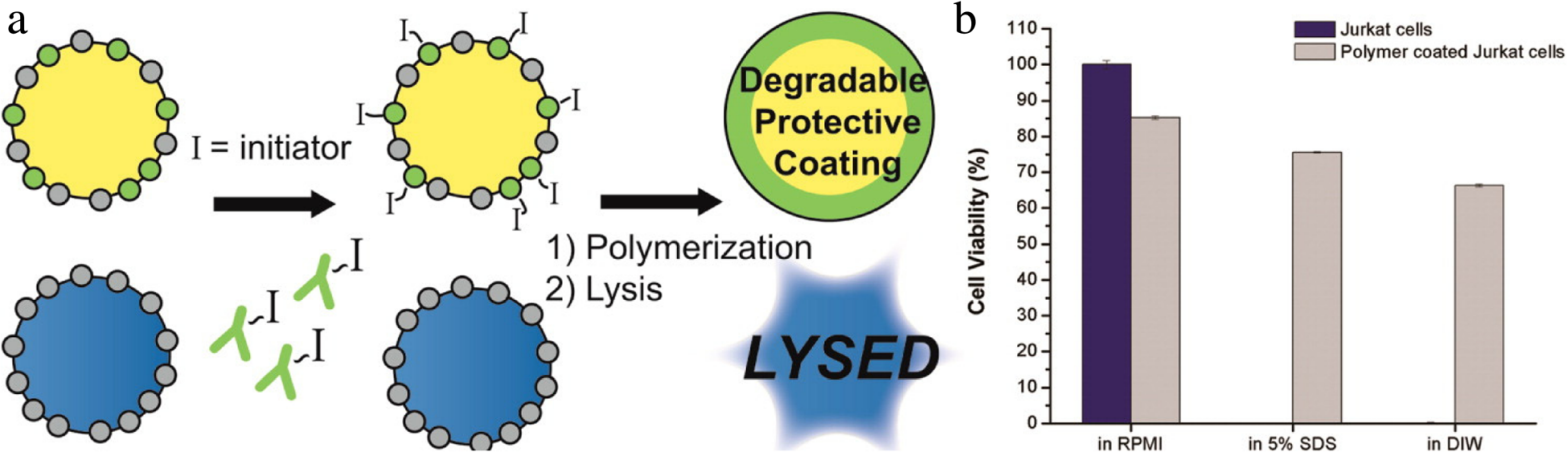
(**a**) Cell selective modification with polymer coating to create protective coating. (**b**) Survival rate of polymer coated cells versus non-coated Jurkat cells. Reprinted from [86], Copyright 2015, with permission from American Chemical Society (https://pubs.acs.org/doi/abs/10.1021%2Facsami.5b06298)

In contrast with MACS and FACS which trade off speed for purity, our lab is developing a new coating-based cell sorting method designed for both speed and purity. In Antigen Specific Lysis, antibodies and other specific interactions are used to specifically mark the target antigen-positive cells with a photoinitiator. The mixture of labeled and unlabeled cells is added to a monomer formulation and irradiated. The exclusive presence of the initiating species at the surface of specific cells localizes the polymerization reaction, forming a nanoscale coating only on the surface of targeted cells. These coatings are designed from derivatives of PEG diacrylate to protect the cells against surfactant and hypotonic lysis. The polymer provides a protective shell during lysis and is later degraded to allow cell proliferation (Fig. 10) [86, 87]. The degradable polymer coating is capable of achieving high Jurkat cell viability in media and when exposed to 5% SDS or deionized water [86].

In the isolation of A549 lung cancer cells from an excess of Jurkat cells, antibodies against EpCAM were used to target the photoinitiator to the A549 cells. Following lysis in 5% SDS a > 98% pure population of A549 cells was achieved. In control studies, antibodies against CD45 were used to isolate a > 96% pure Jurkat population from a majority of A549 cells [86]. This system is also capable of achieving high purity (> 99%) when isolating A549 cells from blood. Coating polymerization and lysis process are batch processes, easily accomplished in under an hour. The batch size is fundamentally limited by delivery of light for polymerization, so scale up is governed by optics, and instruments capable of sorting 10^9^ cells/h are commercially available for approximately $2000 (Photon Systems Instruments). Thus, ASL is a coating approach to cell isolation that delivers high purity and high speed. Product yield is the most significant limitation to this coating-driven isolation process. ASL yield is directly correlated to the density of photoinitiators on the surface of the cell [87]. Cells with low antigen densities result in low photoinitiator density and low cell yield. As a result, the protective coating based ASL isolation is only appropriate for high throughput, high purity isolations where the low yield is tolerated.

## Coatings design

The rational design of a cell coating for a specific application requires consideration of function of the coating and the interaction of the coating with the cell and the local environment. Here, we systematically detail the coating design process moving from the natural cell surface outward. First, the nature of the coupling between the cell and the coating is critical to both the function of the cell and the coating. The coating must support the cell’s natural functions while being anchored to the cell surface. Next, we discuss the impact of coating material selection on cell function and target application.

### Design of Cell/coating interaction

**Fig. 11:**
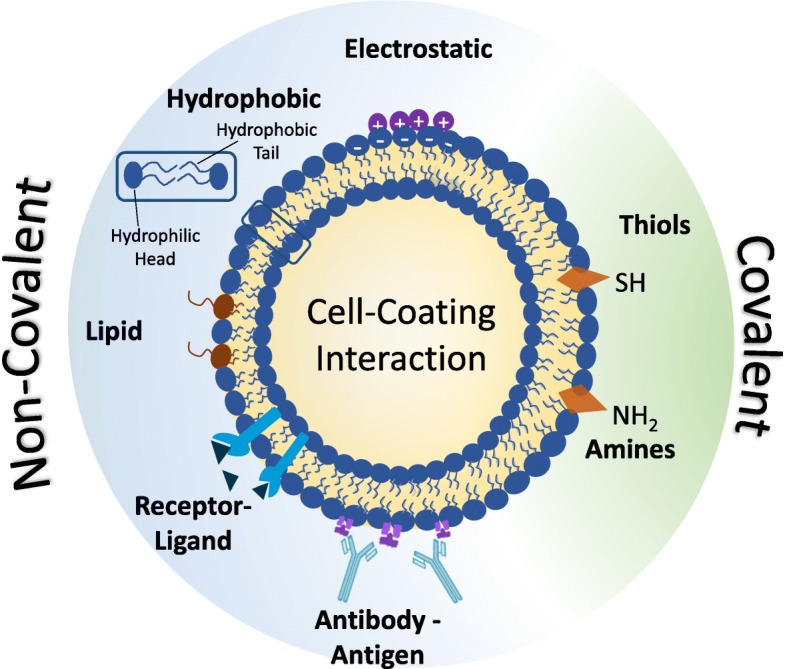
Common methods of anchoring a coating to the mammalian cell surface

The cell coating community benefits from several well-developed bioconjugation techniques that have been developed for the broader concept of cell surface engineering. These are categorized into covalent and noncovalent strategies (Fig. 11), and are extensively reviewed elsewhere [96, 97]. In this section, we specifically discuss the conjugation methods as they relate to the design of a cell coating, and we emphasize the advantages and disadvantages of each technique. Many additional resources are currently published for greater detail on chemical modification of cell surface in the absence of a synthetic coating [96, 98–101].

#### Antibody conjugation

Cell surface proteins act as convenient functional handles to engineer a cell’s surface. Antibodies are commercially available for most proteins found on mammalian cells. Antibodies localize on the cell surface by antigen attachment and can then be further modified to allow polymers or nanoparticles to attach to these antibodies functionalizing the cell surface.

Biotinylated antibodies are commercially available and are commonly used for the attachment of cell coatings. Avidin, streptavidin, and neutravidin all have a high affinity for biotin, and this creates an adaptable linkage between a coating technology and cell surface binding antibodies [59, 86, 87]. For example, Anselmo et al. used biotinylated antibodies to anchor drug loaded patches on monocytes for cell-directed cell therapies. Their “cellular backpack” approach uses layer-by-layer (LBL) fabrication with a magnetic NP-loaded layer for drug payload and an attachment layer terminated with biotin groups that was exposed to streptavidin followed by an antibody of choice allowing for monocyte attachment. Patch-adhered, purified monocytes were then released from the solid substrate to yield monocytes with a LBL coating on one side of the cell [59]. Our lab also uses target specific biotinylated antibodies for cell modification. Biotin, localized on the cell surface, is bound to streptavidin conjugated eosin that when exposed to green light in a monomer mixture, creates a polymer hydrogel coating around the cell [86, 87, 102]. In a distinct approach, some researchers are modifying the lysine residues on antibodies to create antibody conjugates with the coating initiators. Cell selective encapsulation was accomplished by Sakai et al. using antibodies covalently conjugated to horseradish peroxide (HRP). HRP serves as a catalyst in a cross-linking monomer solution to form a hydrogel sheath on the surface of a cell presenting the target antigens [103].

The greatest advantage to antibody anchoring is the ability to target the surface of specific cells. Antibodies only bind to specific antigens, and this selectivity guides the attachment to different types of cell surfaces. This advantage is highlighted in our group’s use of antibody-directed cell coatings for rapid cell isolation [86, 87] and the cell-selective encapsulation achieved by using antibodies conjugated with an HRP catalyst [103]. The most significant disadvantage to an antibody anchoring to the cell surface is the cost of antibody production. Antibody production is done either in vivo or in vitro, where amount of antibody produced is the largest factor in cost. In vitro production on an industrial scale can cost anywhere from $45/mg to $1/mg depending on batch size [104]. The price gets significantly higher for antibodies intended for use in human subjects or custom antibody production [104].

In all, antibodies are often the most convenient choice for the small-scale research and development of cell surface anchoring systems. Antibodies support greater cell viability than most covalent anchoring systems. However, antibody cost will play a major role in the overall economics of the application. It is likely that for most commercial or clinical applications, a nonspecific anchoring system coupled with a conventional upstream cell sorting would be more economical than an antibody anchoring system.

#### Electrostatic interactions

The negative charge of a mammalian cell surface helps to drive necessary ions such as potassium or calcium through the cell. The negative charge on cells results from the presence of sialic acid residues on glycoproteins, and the negative charge is also important in preventing cell aggregation [96]. For cell modification, the negative charge on peripheral membranes serves as a convenient electrostatic binding sites for positively charged polymers, polycations, and nanoparticles. Studies have explored many different polycations to modify mammalian cells including poly-L-lysine (PLL) [105], polyallylamine hydrochloride (PAH) [106, 107], and PEI [79].

This electrostatic anchoring strategy was recently exploited to reduce cell agglomeration that commonly occurs in cell therapy and cell processing. One approach was to replace the slight negative charge on the cells with a high density of positive charges for enhanced cell-cell repulsion. By incubating cells in PLL, the polycation species electrostatically adsorbs to the cell surfaces in a speckled cell coating [105]. Since these coatings only partially covered the cell’s surface area, the cells were able to internalize the polymer within a few days and return to normal function. Additionally, minimal cell aggregation was observed in coated cells while uncoated cells formed aggregates. Ribeiro et al. demonstrate coating cells from two bone cancer cell lines and a fibroblast line [105], while other groups have reported electrostatic anchoring of polymers to K562 cells [107] and macrophages [58]. Due to all mammalian cells having a negative charge, electrostatic coupling can be generalized to many cell populations. The most popular use of electrostatic binding of a polymer to the cell surface is in the field of LBL cell coatings [58, 107, 108], discussed in greater detail below.

Electrostatic cell modification is also convenient for nanoparticle coatings. For example, the surface of superparamagnetic iron oxide nanoparticles (SPIONs) was functionalized with positively charged PAH to generate a stable form of cationic magnetic NPs in aqueous solution. The SPIONs could then be coated onto HeLa cells through simple incubation [106]. A similar strategy of electrostatic coating formation of silica nanoparticles has yielded cytoprotective coatings around cells. The primary cell modification is created through an electrostatic interaction between the cell membrane and PEI [79].

**Fig. 12:**
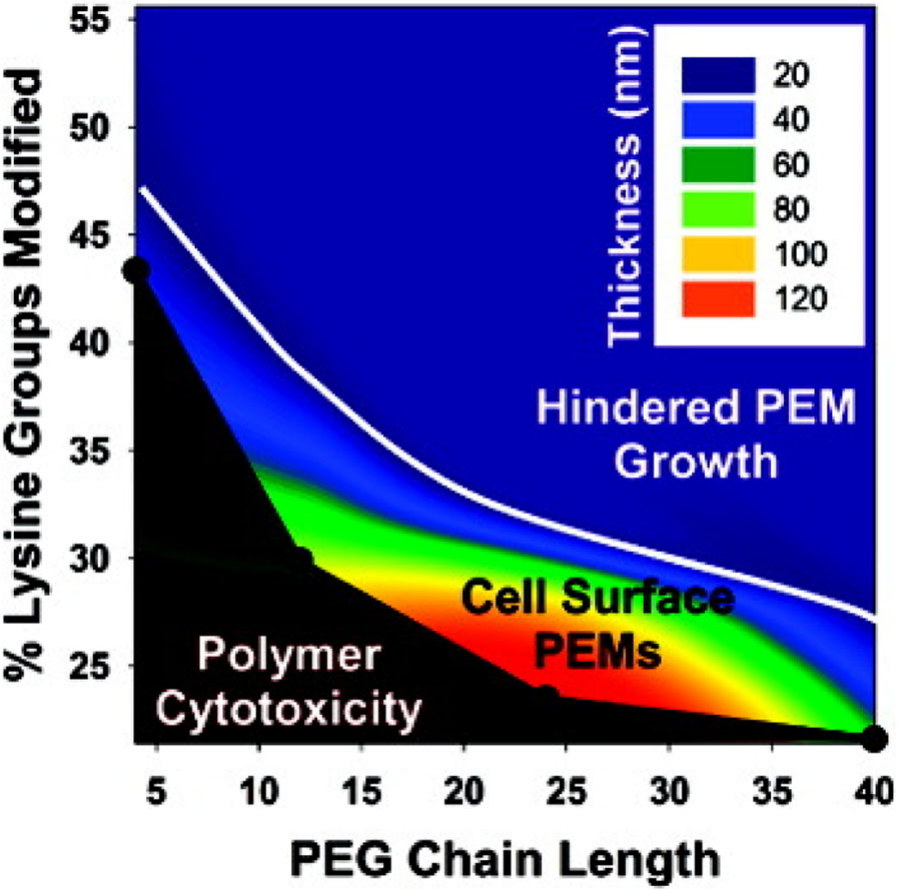
Impact of capping lysine groups with PEG chains on polycation cytotoxicity and the ability to grow multilayer films. Reprinted with permission from [109], Copyright 2011, American Chemical Society

A significant advantage of electrostatic cell modification is that the chemistry involved is simple and inexpensive. Additionally, it is not selective to specific cells making the process easy to translate to different cells or usable in systems with multiple cells types. The promiscuity of electrostatic coupling is a challenge for cell-specific applications. The greatest disadvantage to electrostatic cell modification is the toxicity of many polycations [97, 98]. Polycation toxicity is related to the density of lysine groups and can be mitigated through adequate lysine capping with PEG groups in the polymer backbone (Fig. 12) [109]. Specific polycations also have their own drawbacks, as well. PEI is not degradable and prevents cell growth and division [79], and PLL permeabilizes cells through the formation of polycation pores [105].

#### Lipid and hydrophobic modification

Hydrophobic interactions are crucial in biology as they play a role in lipid bilayer formation and protein folding and interactions [97]. The structure of the phospholipid bilayer in the peripheral membrane creates a stable barrier between the cytoplasm and the environment. By mimicking the hydrophobic structure of the bilayer, it is possible to stably link unnatural components to the cell surface [97]. Given the abundance of phospholipids in the peripheral membrane, modified lipids are rational choices for bilayer integration. Lipid conjugations can be formed through covalent binding of polymers and proteins to the lipid [96]. These lipid conjugates are readily incorporated into the bilayer and have minimal impact of cell viability and function.

CXC chemokine receptor 4 (CXCR4) plays an important role in recruiting MSCs to chemokines released at the site of cardiac injury, including SDF-1. DMPE lipids were used to anchor a conjugate of DMPE-PEG-CXCR4 on the surface of MSCs through hydrophobic interactions within 2 min. The lipid conjugate had no impact on other adhesive molecules, cell viability, or proliferation, and nearly all MSCs had CXCR4 on the surfaces [36]. Lim, K.S., et al. also studied DMPE-PEG-peptide conjugates on MSCs to promote adhesion between the CRPPR peptide and the CRIP2 protein receptor. Jurkat cells were also modified with DMPE-PEG-SPION-FITC to show cells could be modified with tracking agents [110].

Cholesterol is another naturally-present material which resides in the peripheral membrane. Zhang et al. created drug-loaded, multilayer coatings anchored to the cell surface with cholesterol. A uniform coating was formed on individual cells that resulted in controlled release of curcumin that protected cells from reactive nitrogen and oxygen species while maintaining high cell viability [89].

While the attachment of a lipid enables robust anchoring, similar surface modification is attainable with a single fatty acid chain. Palmitoylation is routinely achieved by covalently attaching a palmitate group to the coating material to conjugate the coating to the peripheral membrane [97]. Palmitated protein G (PPG) was mixed with MSCs to allow fatty acid insertion into the cell membrane through lipid-lipid interactions, and protein G was presented on the cell surface. [31]. MSCs have also been modified by Armstrong et al. utilizing a protein-polymer-surfactant complex [111]. Enhanced green fluorescent protein and myoglobin, which promotes tissue oxygenation, was coupled with N,N-dimethyl-1,3-propanediamine (DMPA) where the hydrophobic nonylphenyl tail allowed for high membrane penetration. In a cartilage model system, the myoglobin complex is tight and non-linear allowing for high tissue oxygenation and reduced hMSC necrosis at the center of the cartilage from 42 ± 24 to 7 ± 6%.

The greatest advantages to lipid/hydrophobic cell modification are that 100% protein transfer can be achieved [97] and that it is less harmful to cells compared to covalent modification [96]. Additionally, palmitoylation is a reversible process [97]. However, cholesterol conjugates are difficult to create and require many chemical addition steps [97] and the interactions are not always strong.

#### Receptor-ligand affinity

Surface receptors offer a natural target for the modification of a cell’s surface. Hyaluronic acid (HA) selectively binds to CD44 receptors on lymphocytes and has been utilized as an anchoring strategy in a variety of LBL cell coatings. These LBL coatings are first formed on a solid substrate with a terminal chitosan-HA complex layer. CD44 on the lymphocyte surface binds to the HA, and then the coated cell is released by cleaving the coating from the solid support [56, 57]. This strategy was used to attach HA-coatings to B-lymphocytes, and the cell-coating association is strong enough to resist mechanical separation [56]. Swiston et al. also used an HA-CD44 attachment approach to coat B-lymphocytes and T-lymphocytes with cellular patches, and determined the coating did not impact on CD44 dependent functions or cell migration in uncoated regions of the cell [57]. HA-CD44 affinity was also leveraged by Gilbert et al. to create oriented microtube coatings on lymphocyte B cells to prevent cell-cell interactions by selectively functionalizing the end of the microtube with HA [112].

Cell modification using receptor-ligand interactions allows for cell specific coatings to be created. Additionally, the cell surface is not robustly modified with receptor-ligand approaches [56]. As a potential challenge, the promiscuity of these systems can occasionally lead to uncontrolled aggregation of coated cells [56].

#### Covalent binding

Covalent binding is one of the most common methods of cell coating attachment. Proteins, carbohydrates, and lipids on cell surfaces contain functional groups that serve as covalent binding sites for materials. In contrast to the techniques for generating non-natural functional groups on the peripheral membrane through biosynthetic or metabolic pathways [97, 113], this review focuses only on the naturally-available binding sites on the cell surface. The most commonly used, naturally-present functional groups are amine derivatives and thiols found in cell surface proteins.

In all, covalent cell modification offers a great advantage due to the high binding strength and stability [97]. However, covalent bonding generally is non-reversible [96]. Additionally, many covalent coating strategies have a significant, concentration-dependent decrease cell in viability [114].

##### Amine groups

Amine groups found in lysine groups and at the N-terminus of proteins are easily coupled to carboxylic acid containing polymers and nanoparticles. The most common form of amine group modifcation is through the N-hydroxysuccinimide (NHS) activation of a carboxylic acid. A sulfonated derivative of NHS (sulfo-NHS) is frequently used in cell surface midification for improved water solubility and minimal cellular internalization [97]. Sulfo-NHS chemistry successfully biotinylated a surface for eventual coating with sLex ligands to increase the rolling interaction of the cell with p-selectin [29]. Our lab also used a similar sulfo-NHS-biotin to covalently modify cell surfaces with biotin to later bind a streptavidin-photoinitiator conjugate. Our approach facilitated the eventual attachment of PEGDA cell patches to photoinitiator-labeled A549 cell surfaces through surface-initiated photopolymerization [114]. The PEGDA films were of thickness large enough to load nanoparticles, and sulfo-NHS-biotin concentrations in buffer below 2 mM did not impact cell viability [114]. Covalent binding for amine group targeting has also been employed to enhance drug delivery. Sulfosuccinimidyl-6-(biotinamido)hexanoate can easily be bound to cell surfaces through amine binding. Once conjugated to the cell surface, biotin acts a binding site that allows for the decoration of hMSCs and HUVECs with NeutrAvidin-coated nanoparticle patches [70].

Beyond NHS, polymers are also covalently bound to amines on the cell surface through cyanuric chloride activation. Cyanic chloride covalently links to amines on the cell surface. This method is frequently used owing to its adaptable chemistry and the chemical stability of cyanic chlroide conjugated proteins [72, 97]. Most notably, RBCs were PEGylated by targeting amino acids on the cell surface with cyanic chloride [72].

Alternatively, succinimidyl valerates (SVA) activate carboxylic acids for amide bond formation, and this strategy effectively masked antigens on an erythrocyte surface with high cell viability using an mPEG-SVA conjugate [71, 73]. Similarly, amine-reactive succinimidyl succinate (SS) is effective in amide bond formation for antigen masking with a hyperbranched polyglycerol (HPG). As expected, the density of grafted HPG groups increased with the grafting reaction time [74].

Gołąb et al. provided the only direct comparison of the SVA and NHS conjugation approaches for mammalian cell coatings. They biotinylated Treg cells with either biotin-PEG-SVA or biotin-PEG-NHS. Treg cells, used as an immunosuppressive agent, were coated onto pancreatic islets. Both NHS and SVA allow covalent binding to cell surfaces through primary amines. Over 40% more Treg cells were attached to islets when using SVA linkages compared to NHS [115].

##### Thiol groups

Thiol groups from cysteine in cell surface proteins are present from the reduction of disulfide bridges or, less frequently, from naturally occurring free thiols. Stephan et al. showed high levels of free thiols could be found on T-cells, B-cells, and hematopoetic stem cells, but only minimal levels of free thiols were found on RBCs [66]. With high levels of free thiols not being found in all cells lines, this leads to amino groups being more commonly used due to their high presecence and ease of modification. When thiol groups are avalailable they still serve as good covlaent modification sites due to their higher nucelophilicity than amino groups [116].

Malemide is commonly used in thiol reactions due to its selectivity and stochimoetric addition [116]. Stephan et al. also studied the use of maleimide-thiol conjugation of drug-loaded liposome nanoparticles to various therapeutic cells. The liposomes contained a maleimide-modified lipid bilayer surface that readily reacts with cell surface thiols allowing for the conjugation of NPs to the cell. These grafted nanoparticles were nontoxic to cells and allowed for enhanced drug delivery of IL15 and IL21 [66]. The same group also studied covalent coupling of maleimide functionalized lipid nanoparticles to the thiol groups of T-cells that could allow membrane permeable drugs to penetrate the immunological synapse. Phosphate inhibitor drugs conjugated to the lipid NPs showed greater T-cell expansion at tumor sites and increased treated animal survival [67]. Altenatively, maleimide-based PEGlyation was studied for antigen masking of RBCs. RBCs alone did not have enough thiol groups mask antigens using a maleimidophenyl-PEG species. However, 2-iminothiolane, which reacts with cell suface protein amino groups, introdcued more thiol groups to promote subsequent maleimide coupling [75].

### Material Design for Bulk Coatings

**Fig. 13:**
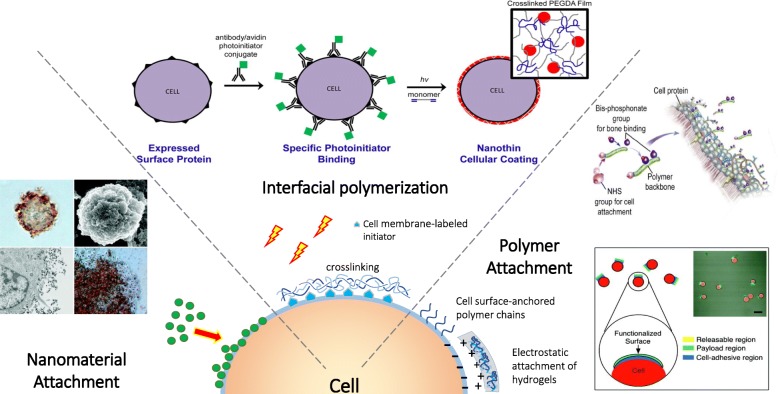
Material systems for coatings on mammalian cell surfaces. Adapted with permission from Langmuir [106], Copyright 2011, American Chemical Society. Adapted with permission from Biomacromolecules [102], Copyright 2015, American Chemical Society. Adapted with permission from Nano Letters [57], Copyright 2015, American Chemical Society. Reprinted from [118], Copyright 2014, with permission from Elsevier

Surface-engineered cells are capable of supporting different categories of adhered bulk materials. The materials on a cell coating can be functionally designed through their structure, surface characteristics, and physicochemical properties for different biomedical purposes. Here, we present the distinct types of materials previously used for cell coatings, including discrete polymer chains, nanoparticles, layer by layer polymers, and interfacial-polymerized hydrogels (Fig. 13).

#### Cell coating composed of discrete polymer chains

In the simplest embodiment, a coating can be constructed from individual polymer chains tethered to the cell membrane. In general, surface-tethered chains are most effective in non-barrier coatings, where the gaps between the chains will not limit the desired coating function. Optimal coating applications for discrete chains include the fluorescent labeling of cell surfaces, cell adhesion, and cell homing. The discrete chains are ideal for these applications as the gaps between chains still permit abundant intercellular sensing and communication. For adequate anchoring and coating functionality, the grafted chains are typically heterofunctional polymer derivatives. In one example, Anderson et al. used a peptide terminated polymer chain grafted to the surface of MSCs for MSC homing to inflamed blood vessels expressing E-selectin [117]. Lower grafting densities of the peptide polymer on MSCs maintained normal viability, proliferation, and differentiation characteristics of uncoated cells. However, at high densities, the coating attenuated the adhesion of MSCs by interfering with adhesion proteins. When compared to crosslinked coatings, the use of discrete polymer chain coatings for this study allows for easier control over surface coverage. The authors were able to identify coating conditions that supported the in vitro adhesion of viable, functional MSCs on the E-selectin modified surfaces under shear flow.

**Fig. 14:**
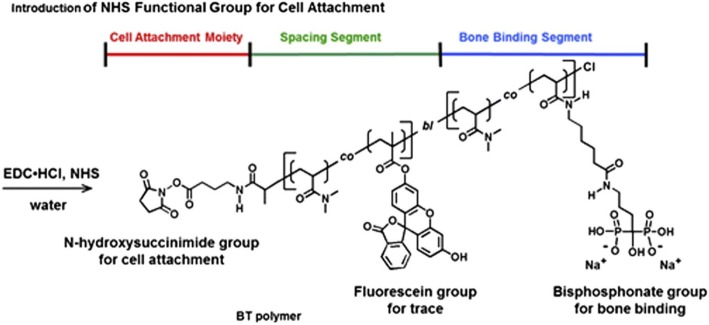
The design of bone-targeted polymer chain attached to the cell surface. One end is for cell attachment and the other end for bone targeting. Reprinted from [118], Copyright 2014, with permission from Elsevier

While heterofunctional PEGs allow the incorporation of two functionalities, controlled radical polymerization is becoming increasingly popular in creating multifunctional polymers for cell surface coatings. In particular, atom transfer radical polymerization (ATRP) offers a simple synthetic route to a diverse range of multifunctional coatings. D’Souza et al. used ATRP to create fluorescently labeled, tissue targeting coatings on HL-60 cells [118]. N,N-dimethylacrylamide was co-polymerized with fluorescein-o-methacrylate to provide a cell-friendly polymer backbone that is easy to track fluorescently. One end of the fluorescent polymer was conjugated with amino-biophosphonate to support bone targeting while the other end was an NHS-activated ester for adhesion to the HL-60 cell (Fig. 14). The coated HL-60 cells exhibited higher interaction with bone fragments in vitro than uncoated cells, while still maintaining high cell viability.

For individual polymer chains to form an effective barrier coating around cells, the chains must be sufficiently dense to prevent interaction with the target species. Most notably, this strategy has been extensively employed in the prevention of alloantigen recognition. Wang et al. grafted a monofunctional SVA-mPEG chain to prevent the identification of antigens on donor cells by host T-cells [71, 73]. Cell PEGylation effectively shielded the surface antigens on human PBMC and murine splenocytes from T-cell recognition [73]. Both the molecular weight and surface density of the grafted polymer chains govern the immunoprotection of mPEG-modified cells. Thicker coatings were observed on the peripheral membranes of PBMCs treated with higher molecular weight (20 kDa) of mPEG when compared to lower molecular weights (2 and 5 kDa), and the concentration of polymer in solution had a positive correlation with coating thickness [73]. Similarly, mPEG is also effective in camouflaging antigens on red blood cells [119, 120].

Discrete polymer chains are also an appropriate strategy for the loading of protective agents into a coating. Incorporation of protective agents into a polymer coating localizes these species to the cell membrane to sequester materials prior to interaction with the cell. Individual cells were coated with a TEMPO-containing poly(dimethylacrylamide) material, and coated cells were protected from oxidative conditions without loss of membrane integrity [90].

The long term stability of discrete polymer chain coatings is dependent on the stability of the surface modification site and the stability of the polymer backbone. Given the discrete nature of the polymer attachment, these coatings are susceptible to internalization coating loss or shedding from extracellular vesicle formation. The timescale of this loss is strongly tied to the attachment site and method, where the activation of internalization pathways will sharply decrease the persistence of the coating on the surface. Most synthetic chains formed via ATRP contain highly stable backbones [118], while peptide based chains will suffer from protease susceptibility [121].

Discrete polymer chains are a highly scalable approach to cell coating deposition. In general, these materials are synthesized at scale, diluted in buffer, and added to the surface of the cell. These polymers are easily manufactured in gram to kilogram batches and are significantly more shelf stable than most therapeutic approaches. The addition of these materials to cells in solution allows the scale up through appropriately size bioreactors.

#### Cell coatings composed of nanoparticles

Discrete nanoparticles are also an effective approach for changing a cell’s surface properties while leaving gaps in the coating for the cell to interact with its environment. The characteristics of nanoparticle coatings closely match those of discrete polymer chains on a surface, but the diversity of coating materials is greatly expanded beyond polymers to metals and inorganic nanoparticles. This expanded material set enables numerous cell coatings applications, including biosensing [34], cell imaging [122], drug delivery [123], and cytoprotection [79]. The nanoparticle dimensions and functional groups regulate cell surface receptor activities and internalization [124, 125]. Recently, the modification of the cell membrane by the immobilization of NPs, as opposed to the potentially cytotoxic intracellular uptake, offers a biocompatible strategy for distinct surface chemical properties for specific cell surface functions.

Cellular coatings based on SPIONs are incredibly useful in cellular sorting and biomedical imaging applications. Dzamukova et al. designed electrostatic cell coatings composed of SPIONs that resisted cellular internalization into the cytoplasm. The positive charged PAH was stably modified on the SPION surfaces by a single incubation step [106]. The PAH-SPIONs were electrostatically deposited onto the negative charged HeLa cells membrane. The coatings had no impact on peripheral membrane completeness, enzyme activity, and cell proliferation. These magnetically-functionalized cells were spatially positioned with a magnetic field.

Silica coatings are offer excellent protection against trypsin and poly(allylamine hydrochloride), these nondegradable coatings are only short-term applications where the cells do not need to proliferate [79]. The silica coatings prevent the normally-adherent HeLa cells from attaching to tissue culture polystyrene flasks. As a potential solution, the silica coating allows for functionalization with 3-mercaptopropyl trimethoxysilane for the deposition of a variety of surface adhesive chemistries [79]. Additionally, the viability of coated HeLa cells seeded into a cell culture flask decreased from of 77% after coating to < 60% after 12 h. The authors conclude the cell division process is hindered by the silica coating, but this limitation could potentially be addressed by tuning the physiochemical properties of the coating. In all, this study illustrates a central compromise with these coatings between short term survival in harsh conditions and long-term proliferation of the cell population.

Polymeric NPs have been extensively studied for the controlled release of drugs. Berlin et al linked polymeric particles to neural stem cells (NSCs) for in vitro delivery to a tumor-conditioned environment [69], and the NSCs displayed selective localization in tumor tissues and were not detected in healthy tissues. Others have arranged these NPs into cellular patches to improve the interactions of the coated cell with its environment during cell-based delivery [70]. These coated cells maintained their tumoritropic capacity, and polystyrene NPs were actively transported to the site an in vitro model of a tumor spheroid. This study also determined that NP aggregation, NP internalization, and peripheral membrane elasticity was strongly dependent on the approach for anchoring these NPs to the cell surface [70]. NP aggregation and internalization is a significant concern for any emerging coating application, and it is especially critical for cell-directed drug delivery applications to ensure these materials are on the peripheral membrane and not in the cytosol of the delivery cell.

The stability of NP coatings is tied to the stability of the NP and the nature of cell grafting. As for discrete polymer chain coatings, NP coatings are potentially lost from the surface by internalization [126, 127], vesicle shedding, or degradation of the NP. Again, internalization and shedding are linked to the chemical nature and location of attachment [125]. Polymeric NPs are typically designed to degrade over well-defined timescales [49], while metallic NPs are often significantly more stable [123].

NPs are also a highly scalable approach to cell coatings. Like discrete polymers, these materials are synthesized separately and mixed with cells in buffer. The maturity of many NP systems enables reproducible manufacturing in large batch sizes, and most NP formulations can be designed for shelf stability. The mixing of NPs with cells allows effortless scale up to clinically relevant doses.

#### Cell coatings composed of layer-by-layer materials

Layer-by-layer (LBL) systems are a powerful and versatile approach for the creation of nanoscale coatings on cells. Most commonly, a polyelectrolyte multilayer (PEM) is formed through the exposure of the cells to alternating charged layers that are electrostatically adsorbed onto the negatively-charged cell membrane. LBL coatings preserve the activity of encapsulated therapeutics, and these coatings are frequently used for tunable drug delivery from multi-drug loaded films [128]. The diverse physical and chemical properties from each selected polymer layer also offer different functionalities for versatile biochemical reactions/functions or mechanical supports in this ultrathin structure. Layers can be polymer-based or particle-based. As a hybrid example, ultrathin PEM layers were directly incorporated onto individual human leukemia cells by LBL electrostatic deposition, and polyhydroxyl fullerene microbeads were loaded into PEM layers to provide adhesion between polyelectrolyte layers. The PEM coating that included polylysine and polyethyleneimine with fullerenol was able to fully encapsulate leukemia cells without inducing nitric oxide production and in vivo inflammatory responses [108].

PEM fabrication allows not only the formation of a complete surface coating but also the creation of polymeric cellular patches. Polymer patches are frequently designed for multiple functional cargos for a variety of potential uses including drug delivery [58, 59, 70, 114], biosensing [56, 57], and control of cell-cell communication [56]. These patches are typically fabricated through lithography on a solid substrate, adhesion of the cells to the patches, and release of the patches from the solid support. This strategy effectively created small thermoplastic circular payloads on cells for as a drug vehicle. The small payload was designed to adhere onto cell membranes and allow unidirectional drug release toward each patch-bound cell through a drug impermeable layers in the patch system. These unidirectional drug release patches are hypothesized to be capable of targeting circulating cancer cells and localizing drug release to only the targeted cells [107].

**Fig. 15:**
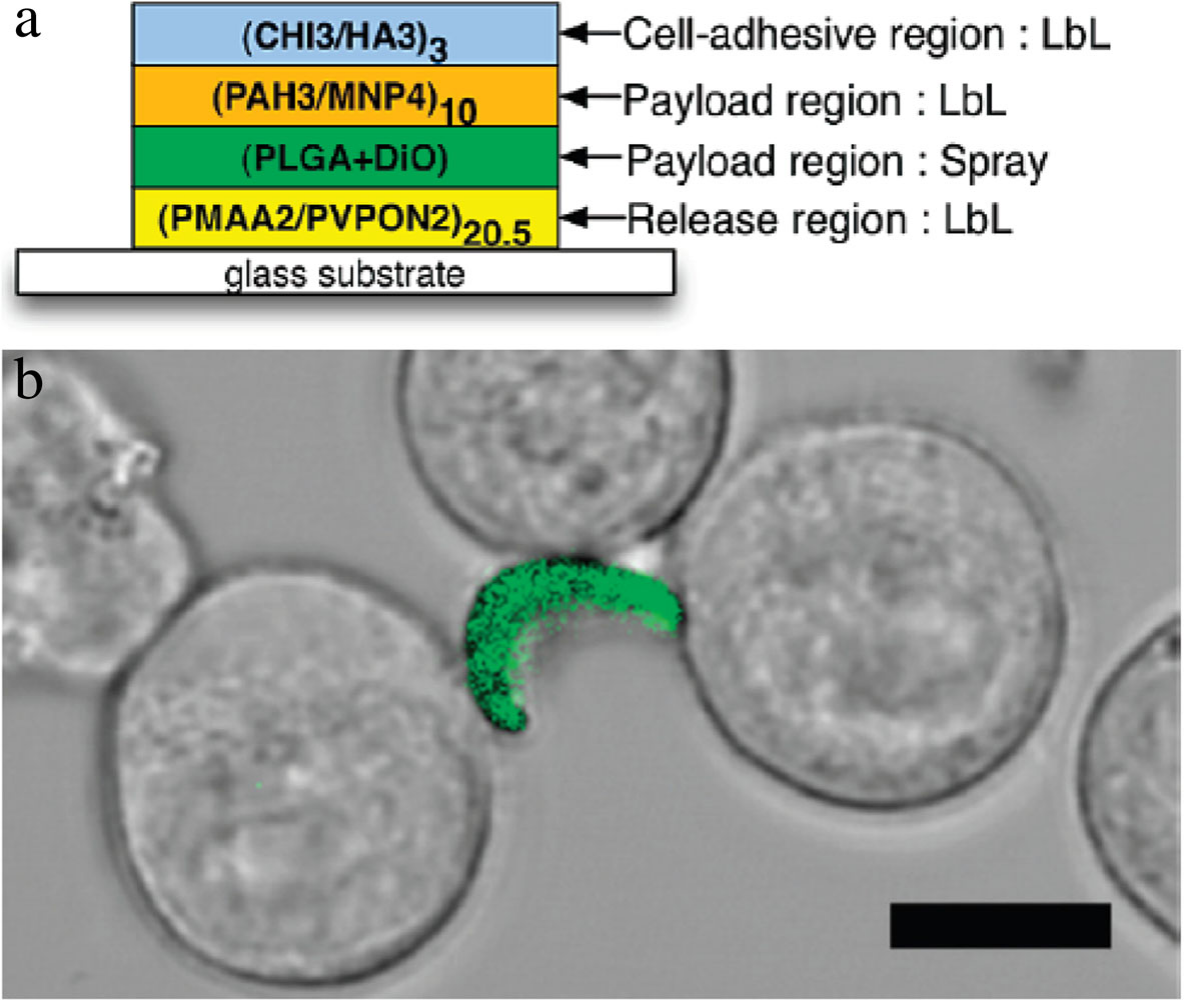
Cell assembly mediated by PEM backpack attachment on macrophage. (**a**) The design of multilayer PEM backpack. (**b**) The backpack attachment was through HA-CD44 interaction on CD44-present macrophage. CHI = chitosan; HA= hyaluronic acid ; MNP= magnetic nanoparticles; PAH= ; PLGA = poly(lactide-co-glycolide); PMAA = poly(methacrylic acid); DiO= 3,3′-Dioctadecyloxacarbocyanine Perchlorate; PVPON= poly(vinylpyrrolidone); PAH= poly(allylamine hydrochloride). Reprinted from [56], Copyright 2010, with permission from American Chemical Society (https://pubs.acs.org/doi/abs/10.1021%2Fbm100305h)

More frequently, LBL cell patches are created with the intention of cell-directed drug delivery, and these “backpacks” of drugs are manufactured by the sequential deposition of polymer release, drug payload and cell adhesion layers on a solid support (Fig. 15a) [56–58, 107]. Release layers between the LBL patch and the solid substrate are either temperature-controlled (with thermo-responsive polymer PNIPAAm) or pH-mediated (with hydrogen-bonding polymer PMAA and PVPONs) dissolution layers. Chitosan/hyaluronic acid-loaded PEM backpacks have effectively labeled CD44 cells through HA-CD44 ligand receptor interaction [56–58]. LBL backpacks also regulate cell communication and aggregation (Fig. 15b). Early work by Rubner et al detailed direct correlations between patch diameter, the number of cells applied on each patch, and the degree of cell aggregation [56]. LBL backpacks on macrophages over 6 μm were sufficiently large to avoid phagocytosis, and cells have negligible changes in mobility and viability after attachment of the cellular backpacks [58]. LBL backpacks were fabricated with PLGA and magnetic NP payload regions to provide drug encapsulation with a capacity of cell tracking through fluorescent or magnetic imaging [56, 57, 59]. These multifunctional coatings were targeted to inflamed tissues through the VCAM-mediated leukocyte migration without obstruction of pulmonary capillaries. Additionally, these LBL backpacks did not interrupt the cell surface receptor interaction in uncoated regions of the cell.

As discussed above, the larger surface area of the LBL coatings frustrates the internalization or shedding of the coatings [58]. These coating lifetimes are typically defined by the stability of the coating substituents. The diversity of LBL coatings substituents enables an extremely wide range of coating lifetimes available, and this attribute is very useful for tuning release profiles in drug delivery applications [59].

Surface adhered LBL processing is amenable to massive production quantities through well-established microfabrication techniques. As such, patches can be readily produced in any desired scale for later attachment to a cell surace. In contrast, the LBL growth of polymer coatings directly on a cell substrate is excessively time consuming. The slow nature of sequential cell incubation and centrifugation steps would require a significant technical advantage of this approach to outweigh the tedious deposition process.

#### Cell coating composed of interfacial hydrogels

Radical polymerization allows a covalently crosslinked coating on the surface of the cell. In general, the crosslinked coatings are formed through a surface-mediated polymerization which restricts the polymerization to the cell surface by only presenting initiation species at the surface of the cell. The immobilization of initiators at the cell membrane is accomplished by covalent binding of cell surface functional groups [87, 102], antibody-antigen affinity [86, 87], or lipid intercalation [129]. Hydrogel films on cells are ideal for applications requiring a completely crosslinked coating, including cell behavioral studies, selective cell isolation [86, 87], and cellular immunoprotection [102].

Hawker et al generated a visible light-mediated hydrogel encapsulation on human Jurkat cells through surface-initiated chain transfer radical polymerization [129]. Their approach used a chain transfer agent coupled to a lipid to link the polymer to the surface of Jurkat cells. Eosin Y was used to initiate polymerization under visible light irradiation, and the growing polymer was grafted to the cell surface through chain transfer. The amount of chain transfer agent on the surface of cells was found to be the main factor in controlling the cell surface polymerization kinetics, and these cells remained viable after hydrogel encapsulation.

**Fig. 16:**
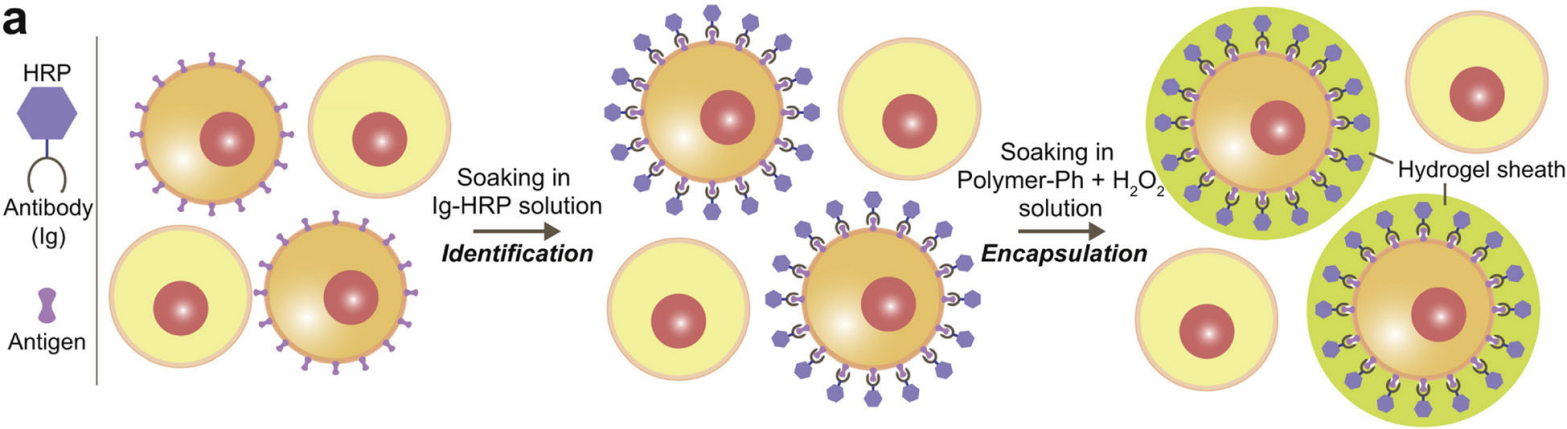
Strategy for enzyme mediated formation of alginate hydrogel films on HRP-labeled cell surfaces. HRP = horseradish peroxidase. Reprinted from [103], Copyright 2015, with permission from Elsevier

Enzymatic polymerization has also effectively formed covalently crosslinked coatings on the surface of cells. Taya et al used HRP as a catalyst to initiate the stable hydrogel crosslinking by attacking the phenolic hydroxyl moieties in the presence of alginate polymer (Fig. 16) [103]. After enzymatic degradation of their alginate hydrogel coatings, the HepG2 cells were able to proliferate at the same level as untreated cells.

Our group combined the concepts of antibody-antigen recognition and free radical photo-polymerization to generate a crosslinked hydrogel only on the surface of antibody-labeled cells. To form these coatings, we adapted a visible light-mediated polymerization scheme developed by Cruise et al. for nonspecific polymerization around porcine islets [130, 131] into a surface receptor-specific cell encapsulation strategy. We restricted the presence of eosin, a photosensitizer used in type II photopolymerization, to the surface of cells using biotinylated antibody labeling and a streptavidin-eosin conjugate. Polymer is only formed on the surface of eosin-loaded cells. Through antigen-specific protection of cells, we created a high throughput cell isolation system. The selection of a crosslinked hydrogel coating supported protection of the cell from both mechanical and chemical insults [86, 87, 102, 114]. The mechanical properties of the covalently crosslinked coating also protected the cell from hypotonic lysis by preventing expansion of the coated cells.

Immunoprotective polymer coatings create design challenges for supporting natural cell function. For the transplantation of therapeutic cells, oxygen and glucose must pass freely across the polymer coating to support cell activity, while the coating must prevent recognition of surface antigens by the immune system. Ultrathin hydrogels with a well-defined molecular weight cutoff maximize flux of beneficial materials while excluding larger species. Our lab has studied the use of surface-initiated polymerization to encapsulate individual Jurkat cells with PEGDA in order to study molecular transport of ultrathin hydrogels [102]. Encapsulation in a 200 nm crosslinked PEGDA film maintained high cell viability and function. Transport of fluorescently-labeled dextrans (4, 10, and 20 kDa) across the polymer cell surface was measured with time dependent fluorescent microscopy. Coatings of polymerized PEGDA-575 and PEGDA-3500 allowed the rapid transport of the 4 kDa species while completely excluding molecules larger than 10 kDa. As expected, coatings formed from larger molecular weight PEGDA species supported more rapid diffusion than smaller molecular weight PEGDA. Of note, the magnitude of the diffusion constants and molecular weight cutoffs of the polymer coatings were comparable to that of the bulk material. The similarity of these transport properties supports a similarity in polymer structure between these nanoscale films and larger bulk materials and simplifies the prediction of coating properties derived from the polymer organization, including transport descriptors as well as coating mechanical properties.

The stability of crosslinked hydrogel coatings is defined by the stability of the bulk hydrogel. As these coatings typically encapsulate the entire cell, the loss of the coatings by shedding or internalization is minimal. The exceptional stability of crosslinked PEG materials create a challenge for prolonged viability of cells, and the programmed degradation these hydrogels through photo- [86, 87] or protease-cleavable [132] subunits is needed. The stability of hydrogels of extracellular matrix components is also tied to the remodeling capacity of the resident cells or the site of implantation [121].

The formation of interfacial hydrogels usually requires the introduction of reaction energy through an irradiation source for rapid gelation and precise control over polymerization times. These light sources present the primary limitation to the scale up of these coating techniques. If adequate light sources are available, these systems can be scaled up readily.

## Characterization challenges unique to coated cells

### Cell analysis

**Fig. 17:**
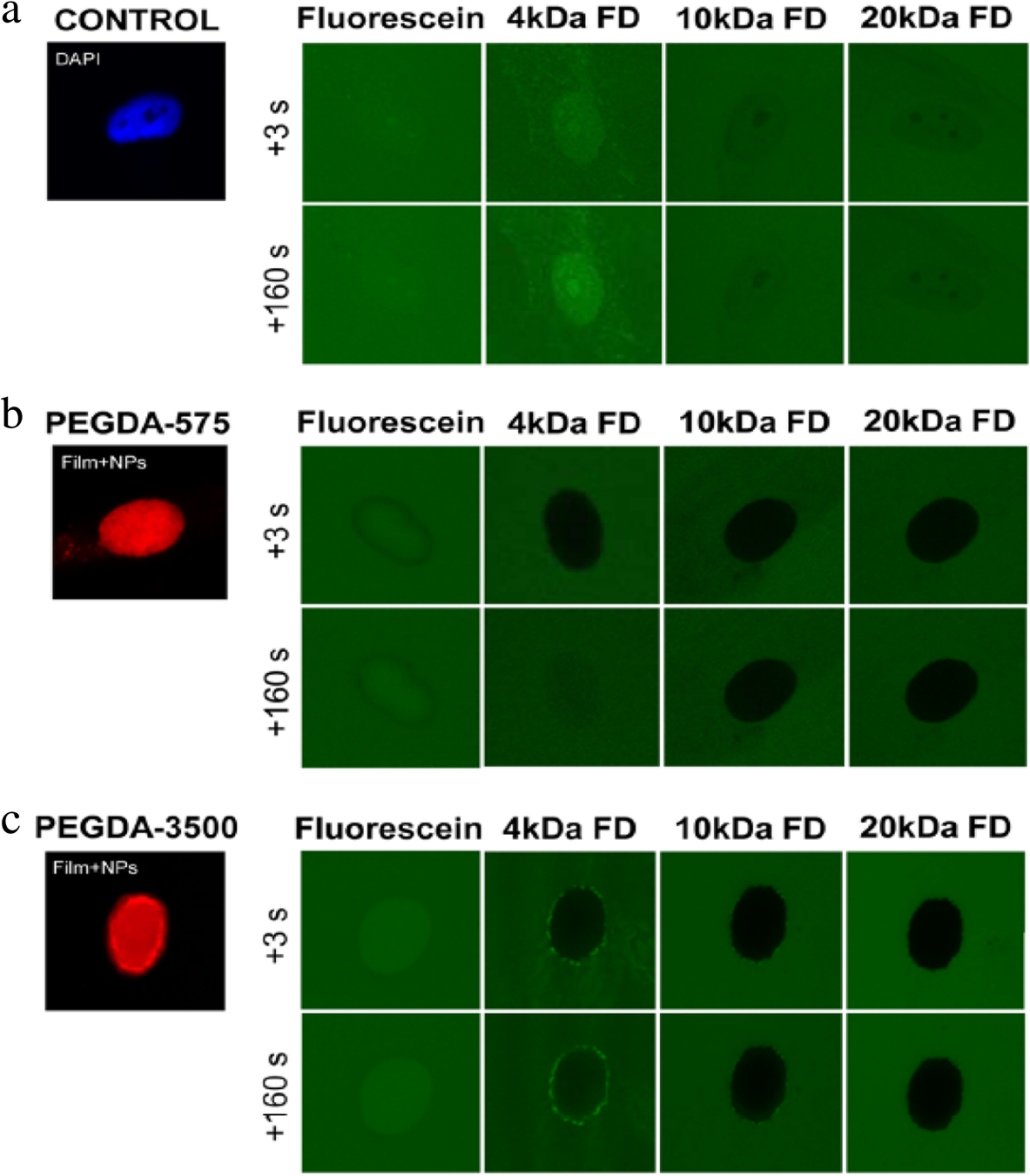
Transport of FITC-dextran in PEGDA-coated Jurkats. The PEGDA hydrogel films prohibit the FITC-dextran larger than 10 kDa. Reprinted with permission from Biomacromolecules [102], Copyright 2015, American Chemical Society

The health and function of the cell is paramount for any coated cell application, and the probes used for viability and function assays must reach the cell for accurate interrogation of function. Critically, the exclusion of these probes by a cell surface coating fundamentally alters the analysis of cellular function. Thus, an explicit description of probe transport across the coating is essential to the assay of coated cells. Our lab has reported that 100 nm thick PEDGA coatings on cells completely prohibit species larger than 10 kDa from interacting with the encapsulated cell [102]. As a result, functional analysis of cell surface receptors using antibodies or moderate sized macromolecular species (fibrobast growth factors, interleukins, transforming growth factors, Tumor necrosis factor alpha) are inconclusive. We also observed slower transport of ~ 4 kDa species across our coatings, suggesting transport of insulin (~ 6 kDa) would be hindered across these PEG coatings (Fig. 17) [6]. As a result, it is reasonable to expect a delayed insulin response to glucose for a healthy, functional B cell inside a hydrogel coating. While other coatings have distinct transport characteristics, this example highlights the importance of membrane transport on these functional assays.

Many viability assays probe the integrity of the cellular membrane using dye exclusion methods [54]. Broken or impaired cells allow colorimetric dyes to label intracellular species (DNA, cytosolic enzyme, mitochondria, etc.). For instance, Trypan blue is a charged dye that stains intracellular proteins only when the peripheral membrane is damaged [133]. Similarly, ethidium bromide is a cell-impermeable, DNA intercalation dye that fluorescently labels necrotic cells [54]. These exclusion assays assume that the cell membrane is the only transport barrier to the permeation of the dye, and any additional transport barrier creates an opportunity for a false-positive viability assay. Similar challenges exist with the transport of probes for intracellular viability analysis. In particular, calcein assays are among the most common viability dyes. For a viable response, the acetomethoxy derivative of calcein must reach intracellular esterases to cleave the acetomethoxy groups to yield fluorescent calcein [134]. Again, the correct interpretation of cell viability depends on prior characterization of acetomethoxy calcein transport across the cell coating. In all, the implications of any transport barrier introduced by the cellular coating must be accounted for prior to any determination of viability and function of an encapsulated cell.

### Quantifying coating thickness

For the thinnest cellular coatings, accurate measurement of the coating thickness is challenging due to optical imagining limits. When dealing with coatings less than 300 nm, surface scientists often use ellipsometry, atomic force microscopy, profilometry and or electron microscopy to determine layer thicknesses. Unfortunately, the irregular shapes and heterogeneous chemical composition of peripheral membrane surfaces introduces significant challenges to get a baseline for measuring the coating thickness. In particular, the carbohydrates extending from the cell surface in the glycocalyx [135, 136] are heterogeneous and are likely intertwined with any cellular coating. Hydrogels also add the extra obstacles to thickness measurement. The solvent choice will dramatically change the measured thickness value. As such, all coating measurements of hydrogel coatings should be in a buffered, hydrated state. The issue of hydrogel solvation precludes all surface analysis tools operating under vacuum and significantly complicates the use of any embedding media. For transmission electron microscopy, the distinction between polymer, cell, and embedding medium is also challenging with coating materials of similar electron densities. When staining these materials, the transport barrier introduced by the coating can also lead to inconsistent staining within samples. As a result, the characterization of coating thickness, and by extension the stability of these coatings, is a significant challenge for the cell coatings community.

Our lab recently used fluorescent intensity correlations as a possible route to address this challenge [102]. Biotinylated bovine serum albumin (bBSA) microarrays on a glass slide were used to mimic a cell surface labeled with biotinylated antibodies. The microarrays were conjugated with streptavidin-eosin and then coated with PEGDA films through a photopolymerization process identical to that used to coat cells. The fluorescence intensity of the microarray using fluorescent NP loaded coatings was measured using epifluorescent microscopy. The coatings on the glass microarrays were measured with profilometery, and the coating thickness correlated strongly with the fluorescent intensity of the image. This correlation was then used to approximate the thickness of the cellular coating based on the fluorescent intensity of the coatings on the cell.

The emergence of super-resolution microscopy is overcoming some challenges associated with coating thickness measurement. Methods such as near-field scanning optical microscopy [137], multi-photon fluorescence [138], stimulated emission depletion [139], and saturated structured-illumination microscopy [140] are capable measuring nanoscale coatings, but often present their own issues such as cell damage or high operation difficulty [141]. Most popularly, stochastic optical reconstruction microscopy (STORM) has been developed as a high-resolution fluorescence microscopy that operates at approximately 20 nm imaging resolution [141]. STORM works by using a photoswitchable fluorophore which favors the off state. As each fluorophore emits a rare emission in the on state, the signal from the isolated fluorophore can be traced to the center of the diffraction limited spot, and the position of that fluorophore is determined with greater resolution than the original diffraction limited spot. Over time, the center of multiple emission events are reconstructed into a fluorescent image with sub-diffraction limit resolution. The ability to measure position within tens of nanometers allows nanoscale cellular coating thickness to be determined. We expect STORM and other super-resolution microscopy techniques to be powerful in the thickness analysis of nanoscale cellular coatings.

### Quantifying ligand surface density

In thin coating formation, the density of linkages to the cells surface plays a significant role in the coating formation process and the ultimate physicochemical properties of the coating. For coatings formed from surface mediated radical polymerization, the abundance of initiator is a significant factor in growth kinetics, and in a quasi-planar geometry this is expressed as a surface density (molecules/μm^2^) [87, 142, 143]. Increased initiator density increases the generation of active radicals near the surface available for polymerization. Using microarrays with serial dilutions of bound initiator we have shown that, while keeping all other parameters constant, thickness of hydrogel formed increases with increasing initiator surface density [144]. We also observed a minimum surface density of initiator for gelation to occur, and each monomer formulation will have its own intrinsic lower limit which depends on many factors such as concentration or monomer molecular weight. For cell surfaces, the abundance of initiator is a significant challenge to complete hydrogel encapsulation.

In efforts to determine specific targets that are suitable for complete coating formation, quantitation of specific surface antigens can be performed [145]. This is typically accomplished by labeling the binding site with a fluorophore, and correlating the fluorescence to a calibrated standard. Our lab used Quantibrite beads to determine the relative abundance of antibody/antigen sites on the surface of a catalogue of human cells and for the nonspecific labeling of amines on the surface of Jurkat cells [87, 145]. In an NHS-based binding system, the number of available amines is significantly higher than is obtained using antibody labeling of the most abundant antigens [87]. At present, there is sparse data on the density of coating binding sites on the surface of cells for cell coatings, and as a result, the implications of grafting density on coating design are yet to be fully detailed.

### Mechanical properties of the coating

In applications of involving physical protection of cells, the quantitative analysis of the mechanical properties of the coating are critical. Unfortunately, the mechanical analysis of materials becomes challenging as the size scale of the material decreases. At small size scales, the contribution of the interfacial structure begins to dominate over that of the bulk material structure, and simply scaling down the bulk properties is not predictive of the behavior of the coating. Additionally, chemical and structural heterogeneity in the material will create significant variation in the local properties, and the impact of this heterogeneity on the target application must be considered.

Direct measurement of the mechanical properties of a thin coating on a cell is challenging. Mammalian cells are highly compliant, and the measurement of a thin, soft hydrogel on the surface of a cell is problematic. For approximation of compressive mechanical properties, micro- and nano-indentation systems are appropriate only if the coating can be accurately recreated on a stiff surface. Tensile testing of the material is only reasonable if it is possible to recreate a thin, unsupported membrane of the material, but the difference between the cell-supported and unsupported interfacial environment must be considered. More commonly, the properties of the coating are approximated by relating the properties of coated cells and the unmodified cells. Here, the cell analysis suite using micropipette aspiration and mechanical indentation developed by Hochmuth and others allows the extraction of cell-associated properties [146].

In analysis of the coating on the cell, there are many important caveats. First, the translation of these cell-associated properties to more common material properties is nontrivial. Cortical tension is a measure of the equilibrium biaxial tension of the cell membrane and associated structures in a spherical geometry. In contrast, most bulk materials are evaluated in a uniaxial tension at a given strain rate in a planar geometry. Next, the natural variance in the mechanical properties of a given type of cell further complicates the mathematics of extracting the coating’s mechanical properties. Finally, cells respond to external stimuli, and any difference in the response of the cell with or without the coating will distort the analysis of the coatings properties. In our own unpublished studies, variable activation of lymphocytes created significant variance in the measurement of the coated and uncoated cells. In all, the mechanical properties of the coated cells are best considered as properties of the cell-coating system and are not quantitatively predicted by the mechanical properties of the bulk materials used in the coating.

## Conclusion and prospects

Cellular coatings fundamentally change how a cell interacts with its microenvironment, and coated cells are being deployed in a rapidly expanding range of applications. Current efforts primarily focus on cell adhesion, cell mediated drug delivery, cellular protection, and cell isolation. Cellular coatings have the potential to impact a broader diversity of fields than those highlighted above. In particular, cellular coatings are ideal for altering cellular building blocks for organoids and tissue structures. New coatings will enable the protection of cells from chemical, mechanical, and biological insults in new synthetic biological tissues and systems. Additionally, coated cells can help direct the assembly of these tissues. Prior work has shown the self-assembly of cells using editing of a cell’s biology [147–149] to have cells of high and low adhesive strength. Similar self-assembly is readily achieved by modulating the adhesive strength of any cell through a cell coating. This would allow core-shell structuring of organoids composed of arbitrary cells without altering the cell’s internal biology.

Coating materials are intimately coupled to their target applications. To date, polymeric cell coatings are largely based on matrices for tissue engineering [6, 7, 102, 114, 118] and layer by layer materials [54–57, 83, 105, 106]. Just as tissue engineering biomaterials are rapidly transitioning from bio-inert to bioactive, the cell coating community is also embracing coatings which deliver drugs [49–52], modulate the immune system [72–75], and naturally degrade [86–88]. The development of responsive and/or naturally degrading inorganic coatings is expected to increase the utilization of coated cells in engineered biological systems. Bioresorbable/bioabsorbable metals are primarily magnesium based, and future cell coating developments would allow temporary metallic coatings [150]. As new functionalities are incorporated into coating materials, the application space for coated cells will continue to expand.

Rare progenitor cells are of high interest across all of biology and medicine. The field of rare cell biology is fundamentally limited by the ability to isolate and study these rare cellular events. While the study of semi-rare tumor cells in circulation is broadly studied, advancements in cell coating yield delivered by more effective surface polymerization strategies will enable the study of more infrequent cell populations. The physiological role of these rare cell populations in many natural and disease states would be possible through technological advancements in cell coatings based isolation.

Characterization tools are rapidly emerging to analyze cellular coatings. Quantitative data on polymer coated cell thickness and uniformity of coverage will be more abundant through the greater availability of super resolution microscopy. Optical resolution of coatings below the diffraction limit of light will give better estimates of the thickness of coatings without correlation to other measured parameters. Additionally, the higher resolution will also improve the detection of small gaps in polymer coatings.

Perhaps the greatest unsolved limitation in polymer coatings is the poor distinction between physisorbed and covalently bound polymers. On stiff 2D substrates, this distinction is determined through rigorous rinsing (shear) or stronger solvents. In the cell coatings, both shear shear and nonaqueous solvents are incredibly damaging to cells. The only possible approach with conventional technology is to directly probe the adhesion of these coatings with optical tweezers, micromanipulation, or atomic force microscopy. The labor intensive direct probing of the coatings would prohibit the collection of the large sample sizes required of biological phenomena. A new technology that can distinguish between covalently bound and physisorbed materials would dramatically improve our ability to develop relationships between the cell-anchoring chemistry and the properties of the coated cell.

The rapid development of functional coatings for single mammalian cells has created an exciting new era of cellular engineering, where the functional design of a cell’s surface is unconstrained by the cell’s biology. Our ability to characterize these coatings and the coated cell are informing the current limitations of these systems while simultaneously empowering the development of new coatings that break these paradigms. As new application ideas drive the development of new coating materials, the complexity of these functional biological hybrid materials will surpass these powerful examples we see today.

## Abbreviations

ATRP: Atom transfer radical polymerization
bBSA: Biotinylated bovine serum albumin
CD: Cyclodextran
CXCR4: CXC chemokine receptor 4
DMPA: N,N-dimethyl-1,3-propanediamine
DOX: Doxorubicin
FACS: Fluorescence activated cell sorting
Fe: Ferric ions
FHP: Ferumoxytol, heparin, and protamine
HA: Hyaluronic acid
HL-60: Human promyelocytic leukemia cells
HPG: Hyperbranched polyglycerols
HRP: Horseradish peroxide
HUVECs: Human umbilical vein endothelial cells
IBD: Inflammatory bowel disease
ICAM-1: Intercellular cell adhesion molecule-1
IT: Iminothiolane
LBL: Layer-by-layer
MACS: Magnetic-Activated Cells Sorting
MAdCAM-1: Mucosal addressin cell adhesion molecule-1
Mal-Phe-PEG: By maleimidophenyl-PEG
MHC: Histocompatibility complex
mPEG: Methoxy PEG
MS: Multiple sclerosis
MSC: Mesenchymal stem cell
NHS: N-hydroxysuccinimide
NP: Nanoparticle
NSC: Neural stem cells
PAH: Polyallylamine hydrochloride
PBMC: Peripheral blood mononuclear cell
PEG: Poly (ethylene glycol)
PEGDA: Poly(ethylene glycol) diacrylate
PEI: Poly(ethyleneimine)
PEM: Polyelectrolyte multilayer
PLL: Poly-L-lysine
PPG: Palmitated protein G
SDF-1: Stromal cell-derived factor 1
SDS: Sodium dodecyl sulfate
SLex: A sialyl-Lewisx
SPIONs: Superparamagnetic iron oxide nanoparticles
SS: Succinimidyl succinate
STORM: Stochastic optical reconstruction microscopy
sulfo-NHS: Sulfonated derivative of NHS
SVA: Succinimidyl valerates
TA: Tannic acid
VBPs: Vascular binding peptides
VCAM: Vascular cell adhesion molecule
VCAM-1: Vascular cell adhesion molecule 1
